# A RACK1 family protein regulates pathogenicity of *Peronophythora litchii* by acting as a scaffold for MAPK signal modules

**DOI:** 10.1080/21505594.2025.2503429

**Published:** 2025-05-13

**Authors:** Guanghui Kong, Rui Li, Weixiong Huang, Yaowen Yang, Tianfang Guan, Jinghan Liu, Wen Li, Tom Hsiang, Pinggen Xi, Minhui Li, Zide Jiang

**Affiliations:** a Guangdong Province Key Laboratory of Microbial Signals and Disease Control/National Key Laboratory of Green Pesticide, South China Agricultural University, Guangzhou, China; b State Key Laboratory for Conservation and Utilization of Subtropical Agro-Bioresources, College of Agriculture, Guangxi University, Nanning, China; c School of Environmental Sciences, University of Guelph, Guelph, ON, Canada

**Keywords:** *Peronophythora litchii*, signal transduction, MAPK, RACK1, pathogenicity

## Abstract

Litchi downy blight caused by *Peronophythora litchii* is the most destructive disease of litchi (*Litchi chinensis*). RACK1 (Receptor for activated C kinase 1) is a group of scaffold proteins, mainly involved in the regulation of various signaling pathways by interacting with signal transduction proteins and affecting the activity of these proteins. In this study, a RACK1 homologue identified in *P. litchii*, and named PlRACK1. The protein was found to interact with the mitogen-activated protein kinases, PlMAPK1 and PlMAPK2. CRISPR/Cas9-mediated genome editing technology was used to knock out *PlRACK1*, and we found that it was involved in mycelial growth, cell wall integrity, ROS metabolism, laccase activity, and pathogenicity of *P. litchii*. PlMAPK1 interacted with RACK1, and they jointly regulated sporangiophore branching of *P*. *litchii*. Transcriptome analysis showed that *P*. *litchii* MAPK Phosphatase 1 (PlMKP1) and beta-glucoside (PlBglX) were regulated by PlRACK1, both of which were also required for the pathogenicity of *P*. *litchii*. As well, PlMKP1 also interacted with PlMAPK1 and PlMAPK2. These results provide insights into the direct interactions between RACK1, MAPKs, and MKP, and their functions in growth, development, and pathogenesis in a plant pathogenic oomycete.

## HIGHLIGHTS

- PlRACK1 played a critical role in response to ROS and pathogenesis of *P*. *litchii*.
- PlMAPK1 interacted with PlRACK1, and they jointly regulated sporangiophore branching.
- PlMKP1 interacted with PlMAPK1/PlMAPK2 and was involved in pathogenesis.
- PlBglX, identified by transcriptome analysis of PlRACK1 mutant, is a virulence factor.

## Introduction

Litchi (*Litchi chinensis* Sonn.) is a subtropical fruit tree belonging to the Sapindaceae and has high nutritional value, attractive appearance, highly valued taste, and high economic value [1]. Litchi downy blight caused by *Peronophthora litchii* (Chen ex Ko et al.) is a major disease of litchi throughout preharvest, and during postharvest, including transportation and storage. It incites rot of tender leaves, flowers, and fruits, and the major preharvest effect is abundant flower and fruit drop, leading to a significant reduction in yield, which can be as high as 80% [2].

RACK1 (Receptor for Activated C Kinase 1) was identified as a protein kinase C (PKC)-binding protein [3]. RACK1 is a seven-bladed-beta-propeller consisting of a tryptophan-aspartic (WD) repeat family [4,5]. Although the WD domain does not have enzymatic activity, it can function in scaffolding to interact with other proteins [6]. RACK1 is a multifaceted scaffolding protein, and the proteins interacting with RACK1 fall into two broad categories: those constitutively bound, and those that are stimulus-dependent or transient. One important functional role for RACK1 is to shuttle some of these binding partners to particular intracellular sites. In addition, RACK1 plays a key role in stabilizing the active or inactive conformation of its partners [7,8]. RACK1 not only binds to three-tier protein kinases in the MAPK cascades to alter their catalytic activity [9] but also interacts with the Gβ subunit of G proteins, linking the G protein signaling pathway to the MAPK signaling pathway [10,11]. In plants, RACK1 positively regulates immune responses in rice (*Oryza sativa*), corn (*Zea mays*), *Arabidopsis thaliana*, or cotton (*Gossypium herbaceum*) [9–13]. In addition, the production of plant hormones, response to stresses, and various growth and developmental processes are also regulated by RACK1 [14].

In fungi, RACK1 was first discovered as part of the 40S ribosomal subunit in *Saccharomyces cerevisiae* and named Asc1, participating in translation regulation [15,16]. In *S*. *cerevisiae*, RACK1 not only functions as a Gβ subunit [17] but also interacts with a member of the p21-activated protein kinase (PAK) family of protein kinases, Ste20, to participate in the Kss1 MAPK pathway [18]. In *S*. *cerevisiae*, the *Asc1* knockout mutant exhibits enhanced sensitivity to cell wall stresses, and its cell size is significantly increased [19]. In *Ustilago maydis*, the RACK1 homologue, Rak1, positively regulates growth, pathogenicity, and lower sensitivity to cell wall stresses [20]. In *Aspergillus nidulans* and *A. fumigatus*, RACK1 plays a crucial role in hyphal growth, spore formation, spore germination, and pathogenicity [21,22]. In *Magnaporthe oryzae*, RACK1 interacts with phosphodiesterase and Gα subunits, regulating the concentration of cAMP, thereby affecting hyphal growth, asexual and sexual reproduction, pathogenicity, and stress responses [23].

Homologues of RACK1 are evolutionarily conserved, and in oomycetes, they have been identified in *Phytophthora sojae*, *P. ramorum*, and *P. infestans* with high protein identity to RACK1 [24]. However, there have been no published reports on the function of oomycete RACK1 proteins.

In various species, RACK1 exhibits a conserved seven internal Trp-Asp 40 (WD40) repeat structure; however, the functions of RACK1 in growth, development, and pathogenesis of oomycetes are still unknown. The purpose of this work was to elucidate the functions of PlRACK1 and its impact on growth, development, and pathogenesis of *P. litchii*, and also investigate potential mechanisms by which PlRACK1 affects pathogenicity. In this study, we found that PlRACK1 was involved in the mycelial growth, sporangial yield, sporangiophore morphology, and pathogenicity of *P*. *litchii*. It interacted with PlMAPK1 and PlMAPK2; and PlMAPK1, similar to PlRACK1, was also involved in sporangial yield and pathogenicity. Furthermore, transcription analysis found that PlRACK1 may regulate PlMKP1 (a MAPK phosphatase interacting with PlMAPK1 and PlMAPK2) and PlBglX (a beta-glucoside), and these two proteins were required for pathogenicity of *P. litchii*.

## Materials and methods

### Strains and cultivation

*Peronophythora litchii* wild-type strain (SHS3) [25], the CK strains, and *PlRACK1*/*PlMKP1*/*PlMAPK1*/*PlBglX* transformants were grown on CJA (juice from 200 g carrot, 15 g agar, and up to 1 L water) at 25°C in the dark [26]. *Escherichia coli* strains DH5α, JM109, and BL21 were cultured on Luria-Bertani agar (LB) at 37°C. *S. cerevisiae* strain AH109 was cultured on Yeast Peptone Dextrose Agar (YPDA) at 28°C. All of these strains have been maintained in our lab.

### Analysis of sporangium, zoospore, and oospore development

To obtain sporangia, 9-mm-diameter hyphal plugs from 5-day-old CJA cultures were each gently washed with 2 mL of ddH_2_O. The resulting suspensions were then filtered through sterile 100 µm-mesh strainers to remove hyphae. The sporangial suspension was incubated at 4°C for 1 h, and subsequently at 25°C for 1 h to release zoospores. Cysts were obtained by vortexing the zoospore suspension for 30 s. The germination of cysts and sporangia required incubation at 25°C for 2 h and 3 h, respectively. Oospores were obtained after incubation at 25°C on CJA for 10 d [27].

### Plasmid construction

Genes *PlRACK1, PlMKP1, PlMAPK1*, and *PlMAPK2* were amplified from cDNA of *P*. *litchii* with specific primers (Table S1 and S2). The amplified fragments were digested with EcoRI and inserted into pET32a or pGEX-6P-1 for protein expression in *E*. *coli*. The vectors pYF2.3 G-Ribo-sgRNA and pBluescript II KS+ were used for the deletion of *PlRACK1*, *PlMPK1*, *PlMAPK1,* and *PlBglX* using CRISPR/Cas9-mediated genome editing technology [28]. To complement these genes in the corresponding mutants, the fragments including the corresponding left arm, gene, and the right arm were amplified and inserted into pBluescript II KS+. The guide RNAs were designed based on the sequence of the left arm or right arm. All primers used in this study are listed in Table S1. These sequences for primer development were from the genome data of *P*. *litchii* [25].

### Nucleic acid extraction and RT-qPCR assays

Total RNA was extracted from diverse life cycle stages (MY, mycelium; 1,5–48 h post-inoculation, hpi; SP, sporangia; ZO, zoospore; CY, cyst; GC, cyst germination; OO, oospore) of *P. litchii*, utilizing the FastPure Universal Plant Total RNA Isolation Kit (Vazyme, China). Subsequently, the total RNA mentioned above was used to synthesize complement DNA, with the HiScript IV All-in-One Ultra RT SuperMix Kit (Vazyme, China). The transcriptional pattern of PlRACK1 was investigated through reverse transcription quantitative PCR (RT-qPCR) technology using 2×PolarSignal ^Ⓡ^SYBR Green qPCR Mix (MIKX, China). The thermal cycling procedure began with an initial denaturation step at 95°C for 2 min, followed by 40 cycles that included denaturation at 95°C for 30 s, and annealing and extension at 60°C for 30 s. The relative expression levels were quantified by the 2^−ΔΔCT^ method [29]. The experiments were repeated three times, and the specific primers for target genes and internal control (PlActin) are listed in Table S1 [30].

The mycelia were obtained after culturing on CJA medium for 3 d. Sporangia were harvested by flooding the mycelia, which had been cultured on CJA media for 5 d, with sterile water, then filtering the subsequent suspension through a 100-µm strainer. The suspension was incubated with sterile distilled water at 16°C for 2 h for release of zoospores. The zoospore suspensions were shaken with a vortex oscillator to cause the zoospores to shed their flagella, leading to loss of motility, and hence resulting in a cyst suspension. The cysts were cultured at 25°C for 2 h, to obtain germinated cysts. The various infection stages were obtained by soaking tender litchii leaves in the zoospore suspension, and then incubating for 1.5–48 h at 25°C. For oospore collection, *P*. *litchii* strains were inoculated onto CJA medium which was covered by an Amersham Hybond membrane. After 10 d, the aerial mycelia were separated from the medium by removing the Amersham Hybond membrane. After that, oospores were scraped from the medium surface and collected.

### CRISPR/Cas9 mediated genome editing

CRISPR/Cas9-mediated genome editing was performed as described previously [28,31]. In brief, the plasmids pYF2.3 G-Ribo-sgRNA and pBluescript II KS+ vectors of these genes (*PlRACK1*/*PlMKP1*/*PlMAPK1*/*PlBglX*) were co-transformed with pYF2-PsNLS-hSpCas9 into *P*. *litchii* protoplasts using polyethylene glycol [30]. The transformants were screened using CJA with 50 μg/mL G418, genomic DNA, and sequencing assays.

### GST pull-down and western blot assays

The plasmids pGEX-6P-1, pGEX-6P-1-PlMAPK1, pGEX-6P-1-PlMAPK2, pET32a-PlRACK1, and pET32a-PlMKP1 were introduced into *E*. *coli* strain BL21. Subsequently, the transformed bacteria were incubated at 18°C with shaking at 180 rpm in the presence of 0.2 mm isopropyl-β-D-thiogalactopyranoside (IPTG) for 12 h to express GST, GST-tagged PlMAPK1/PlMAPK2, and His-tagged PlRACK1/PlMKP1 proteins. For GST pull-down assays, GST and GST-tagged MAPK1/MAPK2 were incubated with 40 μL GST magnetic beads (Thermo Fisher Scientific) for 1 h at 4°C, and then these beads were washed three times with cold lysis buffer (1 × PBS (pH 7.4), 1% Triton X-100%, and 0.1% protease inhibitor cocktail). Then the beads were incubated with His-tagged PlRACK1/PlMKP1 1 h at 4°C, and then washed five times with lysis buffer. After that, the presence of GST-tagged or His-tagged proteins were assessed by Western blot as described previously [32].

### Yeast two-hybrid assay

The pGBKT7-PlMKP1, or the empty vector pGBKT7, and the pGADT7: PlMAPK1/PlMAPK2, or empty vector pGADT7, were co-transformed into *S. cerevisiae* AH109 [33]. Transformants were selected on drop-out (SD)/−Trp−Leu medium and tested on SD/−Trp−Leu−His−Ade medium. Yeast controls showing either positive or negative interactions were provided by the Matchmaker GAL4 Two-Hybrid System 3 (Clontech).

### Sensitivity to various stress

To investigate the sensitivity of *PlRACK1* and *PlMAPK1* mutants under different stress conditions, hyphal plugs (diameter = 7 mm) of WT, CK, mutants, and complementary strains were placed on Plich agar, and cultured at 25°C in the dark for 7 d. Calcofluor white (CFW) can bind to cellulose in the cell wall of oomycetes and be activated by blue light. Plich medium was supplemented separately with different 20 mg/L sodium dodecyl sulfate (SDS), 350 mg/L Congo Red (CR), 50 mg/L Calcofluor White (CFW), 2 mm H_2_O_2_, 0.2 M NaCl, 0.2 M D-sorbitol, or 0.2 M CaCl_2_. These experiments were repeated three times. Inhibition (%) was calculated as follows: For a specific strain, using the strain itself as the control, the formula for Inhibition (%) was calculated as follows: [(the average colony diameter on Plich agar − the average colony diameter on Plich agar with a stress agent)/the average colony diameter on Plich agar] × 100% [26].

### Pathogenicity assays on litchi leaves and fruits

In this study, litchi leaves were collected from South China Agricultural University. We used a detached-leaf inoculation method. A total of 10 µL of zoospores (10/µL) or sporangia (20/µL) were inoculated onto the abaxial side of 8–10 d old litchi leaves or fruits, followed by incubation at 25 °C for 2 d as previously described [34]. After inoculation, the leaves were stored at 25°C with 80% humidity. Subsequently, we measured the lesion lengths and took photographs. This experiment was repeated three times, with each repetition consisting of three replicates.

### Phylogenetic analysis and protein structure modeling

For phylogenetic analysis of PlRACK1 and PlMKP1 proteins, sequences of PlRACK1 and PlMKP1 homologs were retrieved from NCBI (https://www.ncbi.nlm.nih.gov/). Their conserved functional domains were discerned via NCBI-CDD and SMART (https://smart.embl.de/). MEGA 11 was used to construct a phylogenetic tree with the neighbor-joining method and 1000 bootstrap replicates and further refined through ITOL (https://itol.embl.de/). Homologs from animals were used as outgroups.

SMART (http://smart.embl-heidelberg.de/) was used to identify the functional domains of the proteins involved in this study. SWISS-MODEL (https://swissmodel.expasy.org) was used for three-dimensional (3D) protein structure models prediction.

### Transcriptome assay

Detached expanded leaves 8–10 d old were immersed for 30 min in zoospore suspensions of WT or mutant, and then incubated at 25°C with 80% humidity. These samples were collected 12 h later. Three biological replicates were used per treatment, and each biological replicate consisted of five leaves. RNA-seq was conducted using an Illumina 150-bp paired-end reads. The relative expression levels were analyzed using DESeq2 software [35].

### Laccase activity assays

For the detection of laccase activity, 5 mm × 5 mm hyphal plugs were placed on LBA containing 0.2 mm 2,2’-azino-bis (3-ethylbenzothiazoline-6-sulfonic acid) (ABTS, Sigma-Aldrich, USA), and then incubated in the dark at 25°C for 7 d [36]. The experiments were repeated three times independently, with three replicates per treatment each time.

## Results

### PlRACK1 is up-regulated during early stages of infection and under H_2_O_2_ stress

**Figure 1.**
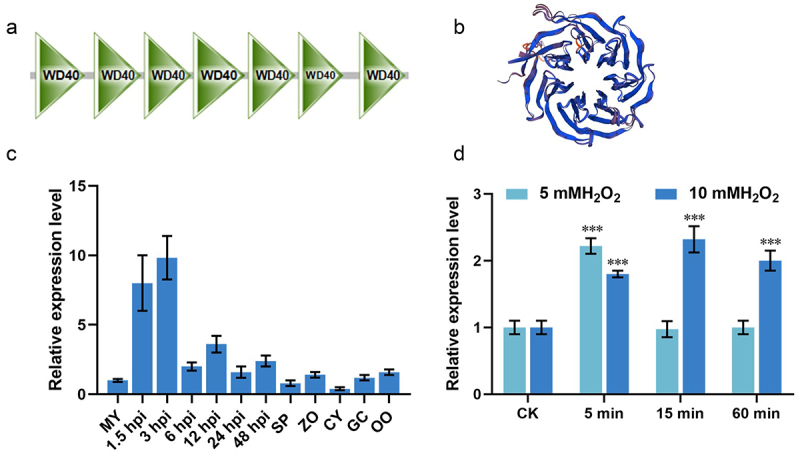
PlRACK1 was up-regulated at an early stage of infection or under oxidative stress. (a) PlRACK1 was predicted to contain WD40 domains through SMART. (b) PlRACK1 three-dimensional (3D) protein structure model generated using SWISS-MODEL (https://swissmodel.Expasy.org/). (c) Expression pattern of PlRACK1 during sexual, asexual, and infection stages of *P. litchii*. Expression levels were determined by RT-qPCR using RNAs extracted from vegetative mycelia (MY), sporangia (SP), zoospores (ZO), cysts (CY), germinated cysts (GC), oospores (OO), and samples from 1.5, 3, 6, 12, 24 and 48 h post-inoculation with zoospores on leaves. (d) The relative expression levels of *PlRACK1* under oxidative stress conditions, 5 mM or 10 mM H_2_O_2_ for 5 min, 15 min and 60 min. Data are mean ± SD. All the experiments were repeated three times independently. Asterisks represent significant differences (Student’s t-test; ***, *p* < 0.001).

In order to identify more pathogenic key genes of *P*. *litchii*, this study focused on the identification and functional analysis of the RACK1 protein and its signaling pathways. PlRACK1 has 317 amino acids (aa) and has a seven-bladed-β-propeller structure composed of seven WD-40 repeats as a conserved RACK1 protein (Figure 1(a,b)). These WD40 domains are located at amino acid sites 5–46, 54–93, 96–135, 137–180, 183–222, 225–262, and 276–314 of the PlRACK1 protein, identified using SMART (http://smart.embl-heidelberg.de/) (Figure 1(a)). Three-dimensional (3D) protein structure models of PlRACK1 generated using SWISS-MODEL had a global model quality estimation (GMQE) score of 0.82, indicating high reliability for classification as a RACK1 protein (Figure 1(b)).

We searched for homologues of PlRACK1 in various organisms including oomycetes, fungi, animals and plants, and used these to construct a neighbor-joining protein tree using MEGA11. Phylogenetic analysis showed that PlRACK1 was most similar to a homolog from *Phytophthora capsici*, and was placed in the same branch with other oomycetes with 100% bootstrap support, but was distant from fungi (Figure S1A).

The expression profile was determined by reverse-transcription quantitative PCR (RT-qPCR) during stages of the *P. litchii* life cycle. Compared with the mycelial stage, *PlRACK1* was up-regulated in the early stages of infection (1.5 and 3 hpi), with 8.24 and 9.86-fold increases, respectively (Figure 1(c)). We speculated that PlRACK1 plays an important role in the early stages of infection, and we further characterized the functions of *PlRACK1* in this study.

Considering that *P*. *litchii* encounters oxidative stress from the host plant in the early stage of infection, we investigated whether *PlRACK1* may be involved in the response to oxidative stress, RT-qPCR was used to assess the transcriptional level of *PlRACK1* under H_2_O_2_ stress in *P*. *litchii*. The results showed that the expression levels of *PlRACK1* were highest (2-fold) for 5 mM H_2_O_2_ after 5 min exposure and highest (2-fold) for 10 mM H_2_O_2_ after 15 min exposure (Figure 1(d)).

### PlRACK1 is associated with mycelial growth, sporangial yield, sporangiophore morphology, and pathogenicity of *P. litchii*

We generated three *PlRACK1* knockout mutants (*plrack1*-4, Δ*plrack1*-15, and Δ*plrack1*-18) and complementary strain (Δ*plrack1*-C) using CRISPR/Cas9 technology (Figure S1B). Genomic PCR assays and sequencing results revealed that *PlRACK1* was replaced by the *NPTII* gene in the three mutants (Figure S1C,D). Subsequently, RT-qPCR analysis confirmed that *PlRACK1* was not expressed in these mutants (Figure S1E). An isolate which had been subjected to *PlRACK1* knockout transformation, but with unsuccessful knockout, was used as a check (CK) strain.

**Figure 2.**
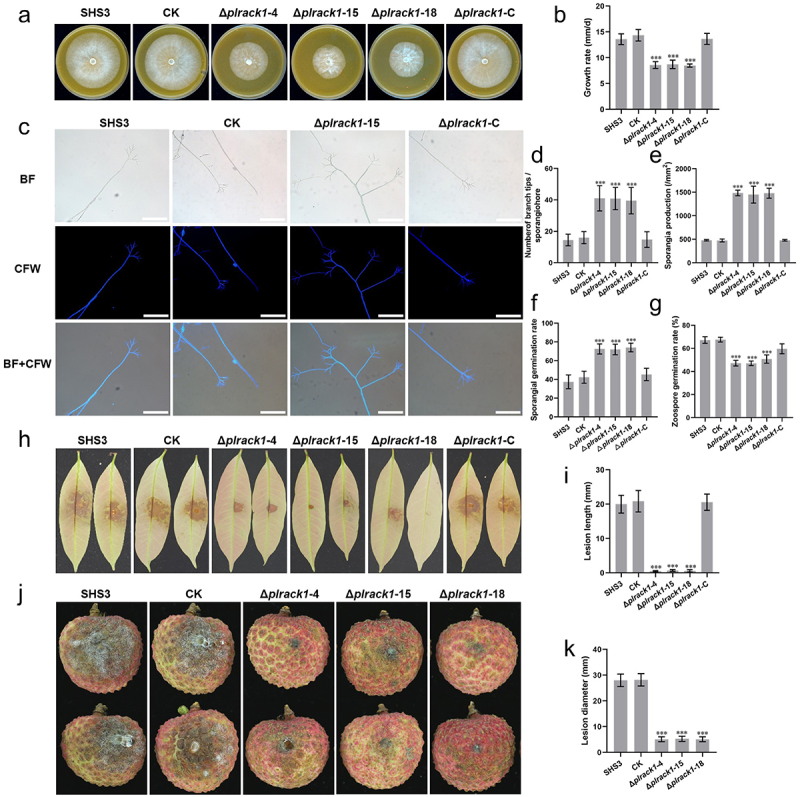
Deletion of *PlRACK1* impaired the mycelial growth, pathogenicity and increased sporangial production of *P*. *litchii*. (A) *PlRACK1* mutants (Δ*plrack1*-4, Δ*plrack1*-15, and Δ*plrack1*-18), WT (SHS3), CK and complementation strains (Δ*plrack1*-C) were cultured on CJA at 25°C in the dark, photographs were taken at 5 d post inoculation (dpi). (b) Growth rates were calculated at 5 dpi according to (a). (c, d) Branches of sporangiophore of *PlRACK1* mutant and WT were observed using fluorescence microscopy (c) and the branch tips were counted (d). Scale bars = 100 µm. (e) Sporangia were quantified at 5 d after inoculation. (f) Sporangial germination rates and (g) Zoospore germination rates of WT, CK, and *PlRACK1* mutants were quantified. (h-k) Pathogenicity test of *P. litchii* RACK1 transformants, WT and CK. (h) Litchi leaves were inoculated with 100 zoospores each of WT and CK, and *PlRACK1* mutants, and then incubated for 48 h at 25°C in the dark. Images show representative leaves for each instance. (i) Lesion lengths on leaves were measured at 2 dpi. (j) Litchi fruits were inoculated with 100 zoospores each of WT and CK, or *PlRACK1* mutants, and then incubated for 48 h at 25°C in the dark. Images showed representative fruits for each instance. (k) Lesion lengths on fruits were measured at 2 dpi. Data are mean ± SD. All the experiments were repeated three times independently. Asterisks represent significant differences (Student’s t-test; ***, *p* < 0.001).

WT, CK, and *PlRACK1* mutants were assessed for mycelial growth rate on CJA after 5 d. Compared to WT and CK, the mycelial growth rates of the mutants were significantly lower (Figure 2(a,b)). To explore the function of PlRACK1 in asexual development, the sporangiophore morphology of PlRACK1 mutants was assessed using light and fluorescence microscopy. We found that sporangiophores of the *PlRACK1* mutants had more branch tips compared to WT and CK (Figure 2(c,d)). Sporangial production, sporangial germination, and zoospore release of WT, CK, and the mutants were analyzed. Compared to WT and CK, the sporangial production and germination by the mutants were significantly higher (Figure 2(e,f)), whereas the zoospore germination rate was lower (Figure 2(g)). Knockout of the *PlRACK1* gene significantly reduced mycelial growth and zoospore germination and increased sporangial production and germination.

To explore the role of *PlRACK1* in the pathogenicity of *P. litchii*, litchi leaves or fruits were inoculated with WT, CK, *PlRACK1* mutants, or the complementation strain, and kept at 25°C for 2 d. We found that the lesions caused by *PlRACK1* mutants were significantly smaller than those of WT and CK strains (Figure 2(h–k)). These results suggested that *PlRACK1* is involved in the pathogenicity of *P. litchii*.

### PlRACK1 is involved in cell wall integrity, tolerance to H_2_O_2_ and CaCl_2_, and laccase activity in *P. litchii*

**Figure 3.**
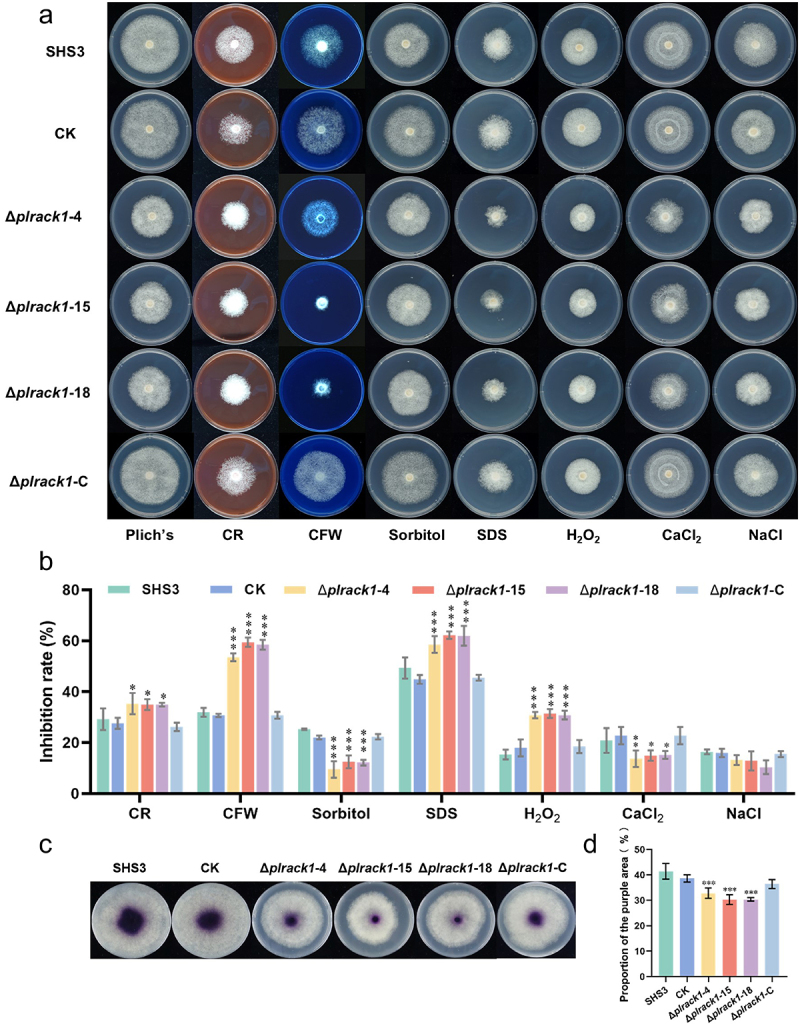
PlRACK1 is involved in tolerance to various stresses and laccase activity in *P*. *litchii*. (a, b) Assay of mycelial growth of SHS3, CK, and *PlRACK1* transformants on Plich medium with/without 350 mg/L CR, 50 mg/L CFW, 0.2 M sorbitol, 20 mg/L SDS, 2 mm H_2_O_2_, 0.2 M CaCl_2_ or 0.2 M NaCl. Images were taken at 5 dpi (a), and rates of growth inhibition were calculated for each treatment relative to growth rate on Plich medium (b). (c) Laccase activity was assessed by observing the oxidation of ABTS to a purple color in LBA media containing 0.2 mm ABTS; photographs were taken 5 dpi. (d) The diameters of oxidized ABTS (dark purple) were measured at 5 dpi. Data are mean ± SD. All the experiments were repeated three times independently. Asterisks represent significant differences (Student’s t-test; *, *p* < 0.05, ***, *p* < 0.001).

To investigate whether *PlRACK1* is related to various stress responses of *P*. *litchii*, the *PlRACK1* mutants, WT, and CK were cultured on Plich agar supplemented with different concentrations of CR, CFW, SDS, CaCl_2_, or H_2_O_2_. Colony diameters at 25°C in the dark were measured after 7 d (Figure 3(a)). *PlRACK1* mutants compared to WT were more sensitive to cell wall stresses caused by CR, CFW, or SDS, as well as oxidative stress caused by H_2_O_2_; but they were more tolerant of sorbitol orCaCl_2_, and there was no significant difference in sensitivity to NaCl (Figure 3(a,b)). These results suggested that PlRACK1 is related to cell wall integrity, and tolerance to H_2_O_2_, sorbitol, and CaCl_2_.

Extracellular laccase activity has been shown to be an important component of microbial defenses against oxidative stresses [37], and hence we analyzed the laccase activity of WT, CK, and *PlRACK1* mutant strains. The isolates were grown on lima bean agar (LBA) amended with 0.2 mm ABTS, and after 7 d, the three mutants had accumulated lower amounts of oxidized ABTS than WT or CK strains significantly (*p* < 0.001), suggesting that *PlRACK1* mutants had lower laccase activity (Figure 3(c,d)).

### RNA-seq analysis reveals differentially expressed genes and metabolic pathways regulated by PlRACK1

Because PlRACK1 plays a crucial role in the pathogenic process, transcriptome analysis of PlRACK1 vs WT was used to uncover the pathways through which PlRACK1 is involved. In the transcriptome analysis of Δ*plrack1*-15 vs SHS3, there were 45,702,558, 45476,838, and 47,637,192 clean reads for the three Δ*plrack1*-15 samples and 38,588,732, 44679,838, and 46,967,320 clean reads for the three SHS3 samples.

DESeq2 [35] was used to analyze differentially expressed genes (DEGs) between WT and Δ*plrack1*-15. The screening criteria for DEGs were |log_2_ Fold change| ≥ 1 and q < 0.05. There were 780 differentially expressed genes among the Δ*plrack1*-15 assembled transcripts compared to WT, where 393 genes were significantly up-regulated, and 387 genes were significantly down-regulated (Figure S2).

**Figure 4.**
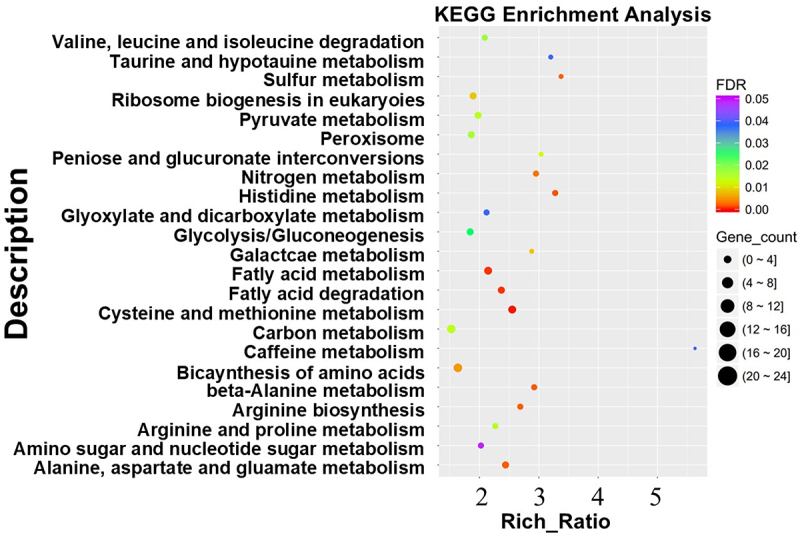
KEGG enrichment analysis of differentially expressed genes. The horizontal axis represents the rich ratio, calculated as (the number of differentially expressed genes in the pathway/the total number of differentially expressed genes)/(the number of genes annotated to the pathway/the total number of genes that can be annotated). The size of the dots represents the number of differentially expressed genes in the pathway. The color of the dots represents the enrichment level of each KEGG entry, with colors closer to red indicating higher enrichment levels.

KEGG (Kyoto encyclopedia of genes and genomes) analysis showed that there were 23 significantly expressed metabolic pathways in Δ*plrack1*-15 compared to WT (Figure 4). GO (Gene ontology) enrichment analysis showed that Δ*plrack1*-15 had 682 up-regulated genes and 697 down-regulated genes in the cellular component; 655 up-regulated genes and 670 down-regulated genes in the biological process; and 394 up-regulated genes and 417 down-regulated genes in the molecular function (Figure S3). Among these results, significant differences in fatty acid degradation pathways were observed in the mutant.

### PlMAPK1 and PlMAPK2 interact with PlRACK1

**Figure 5.**
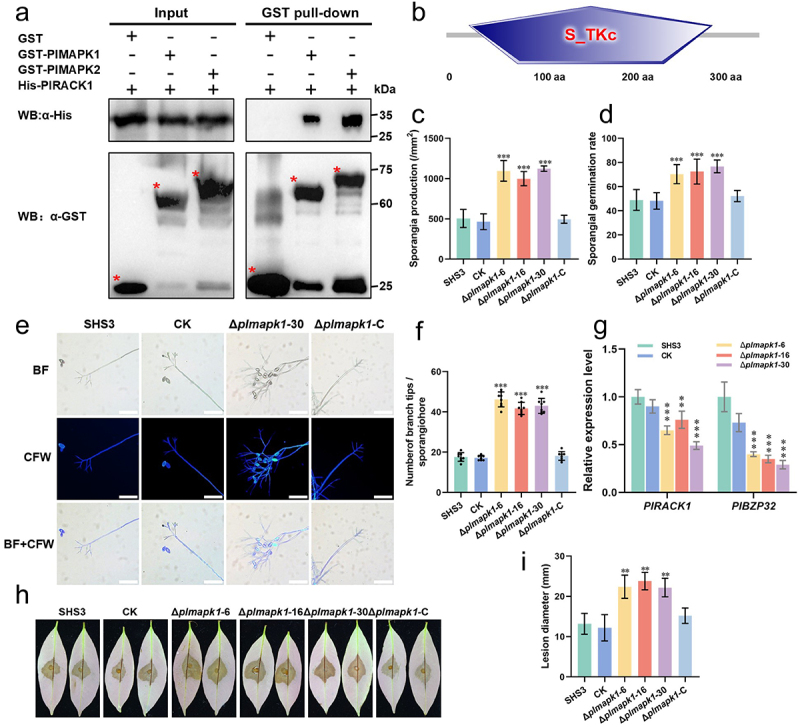
PlMAPK1 interacted with PlRACK1 and is involved in sporangiophore branching and pathogenicity. (a) GST pull-down assays showed that PlMAPK1 and PlMAPK2 were associated with PlRACK1. Western blot was used to analyze the results of GST pull-down assays. GST was used as negative control. (b) PlMAPK1 contains a S_TKc (serine/threonine protein kinase catalytic) domain located at 28–318 aa. (c) Deletion of PlMAPK1 led to higher production of sporangia. Data are mean ± SD of sporangia numbers per mm^2^. (d) Mean sporangial germination rate after incubation for 8 h. (e) Sporangiophore morphology of *PlMAPK1* transformants, WT, and CK were observed and photographed under light and fluorescence microscopy. Scale bars = 100 µm. (f) Mean number of branch tips per sporangiophore. (g) RT-qPCR analysis of the transcription levels of two genes (*PlRACK1*, *PlBZP32*) with increased number of sporangiophore branches in *PlMAPK1* mutants, WT, and CK. The transcription levels of these genes in WT were set to 1. (h) 200 sporangia of WT, CK or *PlMAPK1* transformants were inoculated onto tender litchi leaves and incubated for 48 h at 25°C in the dark. Images show representative leaves for each instance. (i) Lesion lengths were measured at 2 dpi. All the experiments were repeated three times independently. Asterisks represent significant differences (Student’s t-test; *, *p* < 0.05; **, *p* < 0.01; ***, *p* < 0.001).

As a multifaceted scaffolding protein, RACK1 usually binds to three-tier protein kinases in the MAPK cascades to regulate hormones, response to stresses, and various growth and developmental processes among organisms in different kingdoms [11,14], but the function of RACK1-MAPK cascades module in oomycetes was not reported. To further investigate the mechanism of PlRACK1 in development and pathogenesis, we analyzed the interactions between PlMAPK1, PlMAPK2, and PlRACK1 by GST pull-down assays (Figure 5(a)). The results showed that PlMAPK1 and PlMAPK2 specifically interacted with PlRACK1 but not with the negative control, GST (Figure 5(a)).

### PlMAPK1 is also associated with asexual development and pathogenicity of *P. litchii*

We found that PlRACK1 interacted with PlMAPK1 and PlMAPK2. PlMAPK2 is associated with sporangial cleavage and pathogenicity of *P*. *litchii* [38], but the function of PlMAPK1 has not been characterized, and hence we selected it for further analysis. PlMAPK1, a 375-amino acid protein, belongs to the superfamily of serine or threonine-specific kinases. It harbored an S_TKc domain (Serine/Threonine protein kinases, catalytic domain) spanning amino acid positions 28–318, as determined by SMART analysis (Figure 5(b)). We generated three *PlMAPK1* knockout mutants (Δ*plmapk1*-6, Δ*plmapk1*-16, Δ*plmapk1*-30) and one complementary transformant (Δ*plmapk1*-C) using CRISPR/Cas9 technology. An isolate that had been subjected to *PlMAPK1* knockout transformation, but with unsuccessful knockout, was used as a CK strain. PCR assays and sequencing results demonstrated that *PlMAPK1* was knocked out in the three mutants (Figure S4A).

The mycelial growth rates of WT, CK, and *PlMAPK1* transformants (Δ*plmapk1*-6, Δ*plmapk1*-16, Δ*plmapk1*-30, and Δ*plmapk1*-C) were assessed after 6 d of growth on CJA. The growth rates of *PlMAPK1* transformants were similar to those of WT and CK (Figure S4B,C). Deletion of *PlMAPK1* did not affect the germination rate of zoospores (Figure S4D). To identify whether PlMAPK1 is associated to various stress responses of *P. litchii*, the *PlMAPK1* mutants, WT, and CK were cultured on Plich agar supplemented separately with SDS, CR, CFW, H_2_O_2_, sorbitol, NaCl, or CaCl_2_. The results showed that *PlMAPK*1 mutants were more sensitive than WT or CK to stresses caused by 0.2 M sorbitol (Figure S4E,F). On lima bean agar amended with 0.2 mm ABTS, the three mutants accumulated amounts of oxidized ABTS similar to those of WT and CK strains after 7 d of growth (Figure S4G,H). These results suggested that *PlMAPK1* is involved in the tolerance of *P*. *litchii* to osmotic stress.

Next, to test whether *PlMAPK1* regulates the sporangial production of *P. litchii*, WT, CK, and *PlMAPK1* transformants were cultured on CJA for 5 d, and sporangia were counted. Compared to WT and CK, the sporangial production of Δ*plmapk1*-6, Δ*plmapk1*-16, or Δ*plmapk1*-30 was significantly higher than WT or CK (Figure 5(c)). Additionally, compared to WT and CK strains, *PlMAPK1* mutants showed significantly higher sporangial germination rates (Figure 5(d)). Subsequently, the sporangiophore morphology of WT, CK, and *PlMAPK1* mutants was observed using microscopy, and we found that *PlMAPK1* mutants produced more sporangia than WT or CK strains, and each sporangiophore of a *PlMAPK1* mutant produced more branch tips and sporangia compared to WT or CK (Figure 5(e,f)). To further investigate the mechanism of *PlMAPK1* involved in the development of sporangiophore branches, we analyzed the transcription of *PlRACK1* and *PlBZP32*, which negatively contribute to the morphology of sporangiophores [39]. In *PlMAPK1* mutants, the expression levels of *PlRACK1* and *PlBZP32* were significantly decreased (Figure 5(g)).

Finally, to explore the role of *PlMAPK1* in the pathogenicity of *P. litchii*, tender litchi leaves were inoculated with 10 µL sporangial suspension (200 sporangia) each of WT, CK, and the *PlMAPK1* mutants. Compared to WT and CK strains, the lesions caused by *PlMAPK1* mutants were significantly larger (Figure 5(h,i)). Taken together, these results indicated that knockout of the *PlMAPK1* significantly increases the pathogenicity of *P*. *litchii*.

### Transcriptome analysis of PlMAPK1 and PlRACK1 reveals shared pathways in regulating phenotypes of *P. litchii*

To further explore the function of the *PlMAPK1*, we performed transcriptome sequencing on WT and the *PlMAPK1* mutant Δ*plmapk1*-30. In the transcriptome analysis of Δ*plmapk1*-30 vs SHS3, there were 40,134,902, 41105488, and 43,821,442 clean reads for three Δ*plmapk1*-30 samples and 40,813,290, 44194396, and 38,970,598 clean reads for three SHS3 samples. Differential expression gene analysis of Δ*plmapk1*-30 vs WT identified 2603 differentially expressed genes, among which 1360 genes were significantly down-regulated and 1243 genes were significantly up-regulated. This indicated that PlMAPK1 affected the expression of a large number of genes (Figure S5).

**Figure 6.**
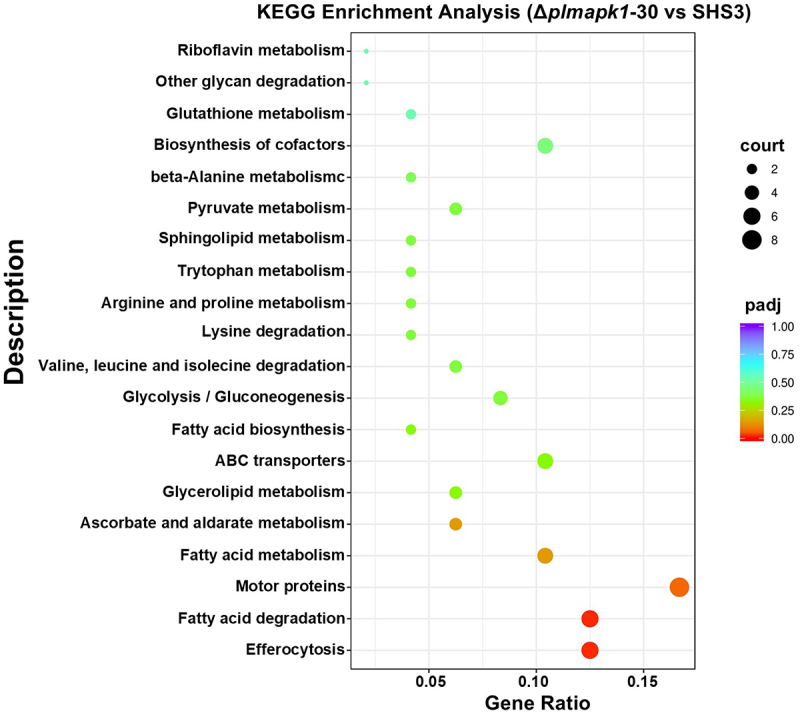
KEGG enrichment analysis of differentially expressed genes in PlMAPK1 mutant Δ*plmapk1*-30 vs WT. The results only show the top 20 metabolic pathways with the most significant enrichment. The horizontal axis represents the gene ratio. The size of the dots represents the number of differentially expressed genes in the pathway. The color of the dots represents the enrichment level of each KEGG entry, with colors closer to red indicating higher enrichment levels.

Both *PlMAPK1* and *PlRACK1* affect the pathogenicity, sporangial production, and the branching of sporangiophores of *P. litchii*. In KEGG analyses of *PlMAPK1*(Δ*plmapk1*-30 vs WT) and *PlRACK1* (Δ*plrack1*-15 vs WT) transcriptome, DEGs were commonly enriched in valine, leucine and isoleucine degradation, pyruvate metabolism, glycolysis/gluconeogenesis, fatty acid metabolism, fatty acid degradation, beta-alanine metabolism and arginine and proline metabolism, suggesting that these pathways may play a key role in jointly regulating these phenotypes (Figure 6).

### PlRACK1 regulates the expression levels of PlMKP1, a MAPK phosphatase

From FPKM, GO, and KEGG results, a *P. litchii* gene for Mitogen-Activated Protein Kinase Phosphatase 1 (*PlMKP1*, *Pl_013853*) was identified, for which the FPKM value was 8.3 in WT and 0 in Δ*plrack1*-15. RT-qPCR assays also confirmed the relative down-regulation of *PlMKP1* in Δ*plrack1*-15 (Figure S6). In the GO enrichment analysis, the protein PlMKP1 was annotated as belonging to the category catalytic activity, and classified as molecular function (Figure S3).

### PlMKP1 interacts with PlMAPK1 and PlMAPK2

**Figure 7.**
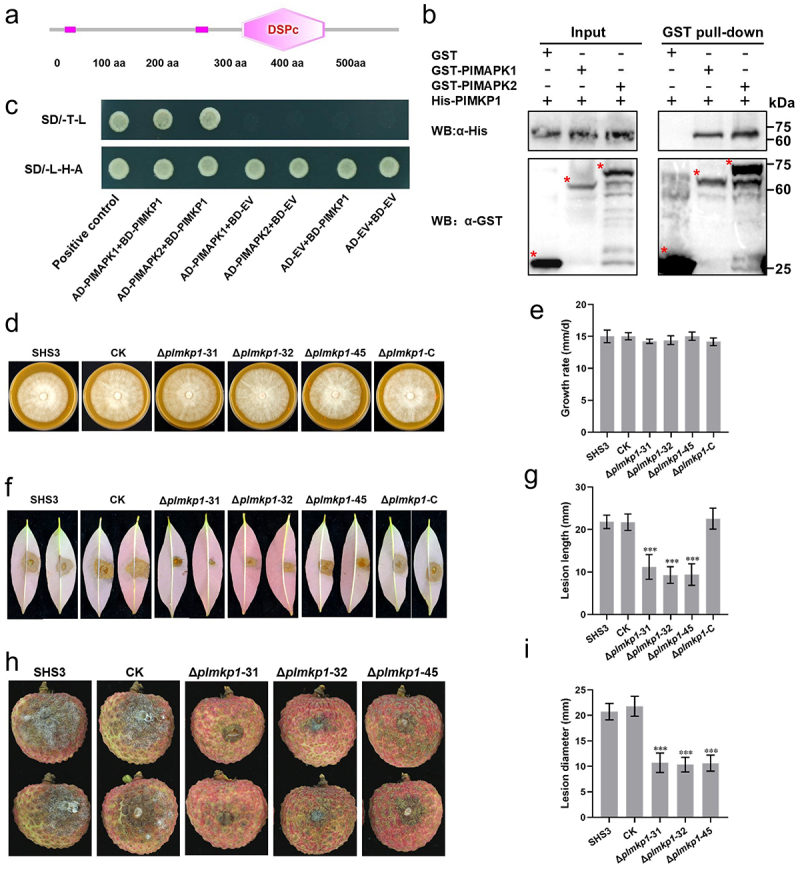
PlMKP1 interacted with PlMAPK1 and PlMAPK2 and involved in the pathogenicity of *P*. *litchii*. (a) PlMKP1 contains a dual specificity phosphatase catalytic domain (DSPc) located at 324–463 aa. (b) GST pull-down assays showed PlMAPK1 and PlMAPK2 associated with PlMKP1. GST was used as negative control. Western blot was used to analyze the results of GST pull-down assays. (c) Yeast co-expressing AD-PlMAPK1/AD-PlMAPK2 and BD-PlMKP1, and the positive control, grew on SD/-Trp/-Leu/-His/-Ade selective medium, while the negative controls did not grow. (d) *PlMKP1* transformants (Δ*plmpk1*-31, Δ*plmpk1*-32, Δ*plmpk1*-45 and Δ*plmpk1*-C), WT, and CK were cultured on CJA at 25°C in the dark, and photographed at 5 dpi. (e) Growth was measured on CJA. (f) Litchi leaves were inoculated with 100 zoospores of WT, CK, or *PlMKP1* transformants, and incubated for 48 h at 25°C in the dark. Images showed representative leaves for each instance. (g) Lesion lengths on leaves were measured at 2 dpi. (h) Litchi fruits were inoculated with 100 zoospores each of WT, CK, or *PlMKP1* transformants and incubated for 48 h at 25°C in the dark. Images showed representative fruits for each instance. (i) Lesion lengths on fruits were measured at 2 dpi. Data are mean ± SD based on at least three replications in each of three independent experiments. Asterisks represent significant difference (Student’s t-test; ***, *p* < 0.001).

PlMKP1 encodes a 588 aa protein. We predicted the protein domain using SMART, and found that the PlMKP1 belonged to a superfamily of protein tyrosine phosphatases and its dual specificity phosphatase catalytic domain (DSPc) was located at 324–463 aa (Figure 7(a)). Sequence analyses showed that PlMKP1 had 83.7% similarity with the MAPK Phosphatase 1 (KAG6586587.1) of *Phytophthora cinnamomi* [40].

To further explore the genetic characteristics of PlMKP1, we searched for homologous PlMKP1 proteins in various organisms including oomycetes, fungi, animals, and plants in NCBI, and constructed a neighbor-joining phylogenetic tree using MEGA11 with 1000 bootstrap replicates. The tree showed that the closest homologue to PlMKP1 of *P. litchii* was from *Phytophthora megakarya* (99% bootstrap support), and both were nested within a clade of *Phytophthora* species with 97% bootstrap support (Figure S7). This clade was separate from plants, animals, and fungi with 100% bootstrap support. In the *Phytophthora* clade, most of the PlMKP1 homologous proteins were predicted to be hypothetical proteins containing dual-specificity phosphatase catalytic domains (DSPc), and only a few of these proteins were fully annotated, such as MAPK phosphatase in *Phytophthora cinnamomi* KAG6586587.1 (Figure S7).

The interactions between PlMAPK1, PlMAPK2 and PlMKP1 were confirmed by GST pull-down assay. The results showed that PlMAPK1 and PlMAPK2 specifically interacted with PlMKP1 but not with the GST control (Figure 7(b)). The interactions of PlMAPK1 and PlMAPK2 with PlMKP1 were also confirmed by yeast two-hybrid analyses (Figure 7(c)).

### PlMKP1 is associated with pathogenicity of P. litchii

We generated three *PlMKP1* knockout (Δ*plmpk1*-31, Δ*plmpk1*-32, Δ*plmpk1*-45) and one complementary (Δ*plmpk1*-C) using CRISPR/Cas9 technology. PCR assays and sequencing results demonstrated that PlMKP1 was knocked out in the three mutants (Figure S8A). Subsequently, RT-qPCR analysis confirmed that *PlMKP1* was not expressed in these mutants (Figure S8B). An isolate that was subjected to *PlMKP1* knockout transformation, but with unsuccessful knockout, was used as a check (CK) strain.

The mycelial growth of WT, CK, and *PlMPK1* transformants was measured 5 d after inoculation. The mycelial growth rates of the mutants were similar to those of WT and CK (Figure 7(d,e)). These results suggested that knockout of *PlMKP1* did not affect mycelial growth of *P*. *litchii*.

Previous results demonstrated that the knockout of the *PlRACK1* significantly reduced the pathogenicity of *P*. *litchii*, but because the expression levels of the *PlMKP1* gene were significantly down-regulated in the *PlRACK1* knockout mutant, the pathogenicity of *PlMKP1* knockout mutants was tested. Zoospore suspensions (10 µL of 10 spores per µL) of *PlMPK1* mutants were inoculated on litchi leaves and fruits, and kept at 25°C. At 2 dpi, we measured lesion length, and found that the lesions caused by *PlMKP1* knockout mutants were significantly smaller than those of WT and CK strains (Figure 7(f–i)). These results indicated that *PlMKP1* is associated with the pathogenicity of *P*. *litchii*.

### Transcriptome analysis of PlRACK1 mutant identified PlBglX, a beta-glucoside associated with growth and pathogenicity of *P. litchii*

**Figure 8.**
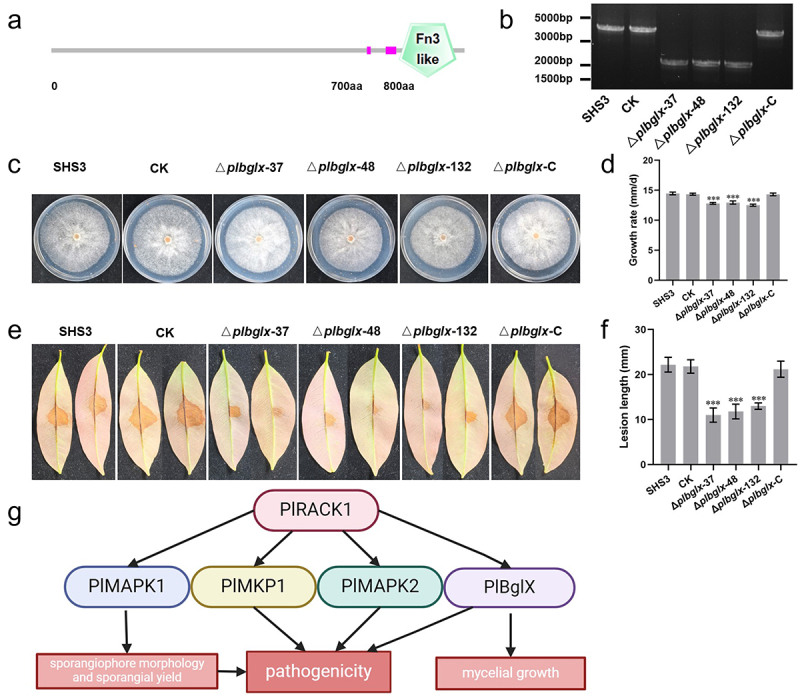
Transcriptome analysis identified *PlBglX* involved in the growth and pathogenesis of *P. litchii*. (a) PlBglX contains a Fibronectin type III-like domain which is located at 789–865 aa. (b) CRISPR/Cas9-mediated deletion and complementation of *PlBglX1*. PCR analysis of the *PlRACK1* mutants. (c) Colonies of Δ*plbglx*-37, Δ*plbglx*-48, Δ*plbglx*-132, and Δ*plbglx*-C, WT, and CK were cultured on CJA at 25°C in the dark for 5 d. (d) Growth rates were measured on CJA. (e) Litchi leaves were inoculated with 100 zoospores each of WT, CK, and *PlBglX* transformants, and incubated for 48 h at 25°C in the dark. Images showed representative leaves for each instance. (f) Lesion lengths were measured at 2 dpi. Data are mean ± SD based on at least three replications in each of three independent experiments. Asterisks represent significant difference (Student’s t-test; ***, *p* < 0.001). (g) The schematic diagram shows the functions of RACK1 and its related signaling pathways, as well as the interaction relationships among RACK1, MAPKs, and MKP1.

In the transcriptome data, we also identified a Pl_000214 sequence, for which the FPKM value was 223.8 in WT, and 75.2 in Δ*plrack1*-15, with approximately 3-fold down-regulation. The Pl_000214 sequence was identified as a beta-glucosidase, and named PlBglX, which encoded an 876 aa protein. We predicted the protein domain using SMART, and found that the PlBglX protein contains a Fibronectin type III-like domain which is located at 789–865 aa (Figure 8(a)).

To explore the role of *PlBglX* in the mycelial growth of *P. litchii*, we generated three *PlBglX* knockout mutants (Δ*plbglx*-37, Δ*plbglx*-48, Δ*plbglx*-132) and gene complementation strain (Δ*plbglx*-C) using the CRISPR/Cas9 technology. Genomic PCR assays and sequencing results demonstrated that *PlBglX* was knocked out in the three mutants (Figure 8(b)). The mycelial growth rates of WT, CK, and *PlBglX* mutants were measured. The mycelial growth rates of the *PlBglX* mutants were lower than those of WT and CK (Figure 8(c,d)).

Zoospore of WT, CK, and *PlBglX* mutants were inoculated onto the abaxial surface of tender litchi leaves, and kept at 25°C. At 2 dpi, we measured the lesion length and found that the lesions caused by *PlBglX* mutants were smaller compared to WT or CK (Figure 8(e,f)). These results suggested that *PlBglX* is involved in the growth and virulence of *P*. *litchii*.

## Discussion

In *Homo sapiens*, *Arabidopsis thaliana*, *Saccharomyces cerevisiae*, and other organisms, RACK1 has been found to participate in the regulation of the MAPK signaling pathway. When the MAPK signaling pathway is activated, that is, when the three-tiered protein phosphorylation of MAPK occurs, RACK1 binds to the MAPK protein as a scaffold protein to assist in phosphorylation and to stabilize the activated state [9,10]. From earlier research in our lab, we predicted 14 MAPK proteins from the genome of *P. litchii* [38]. From the current research, one RACK1 homologue protein, named PlRACK1, was identified in the genome of *P. litchii*, and functionally characterized.

This study verified PlRACK1 as a typical RACK1 protein and that its interactions with PlMAPK1/PlMAPK2 resemble the action of RACK1 in plants [11]. This suggests that the RACK1-MAPK complex might serve as a signaling module and play a crucial part in the life cycles of oomycetes. PlRACK1 and PlMAPK1 jointly modulated sporangiophore development; PlRACK1 and PlMAPK2 coordinately regulated pathogenesis and laccase activity [38]. These findings implied that PlRACK1 and MAPKs could function as a signaling module to jointly regulate development and pathogenesis (Figure 8(g)). PlMAPK2 specifically regulates the cleavage of sporangia [38], indicating that its function is not entirely dependent on its binding to PlRACK1. Further investigation is required to elucidate the detailed biological relationship between PlRACK1 and MAPKs.

*P*. *litchii* mainly spreads through the production of large numbers of asexual spores, namely sporangia and zoospores [41]. The mycelial growth rate of the *PlRACK1* mutant was significantly reduced, and the number of sporangiophore branches increased significantly, resulting in denser mycelia and a significant increase in sporangial production (Figure 2). Furthermore, we found that PlRACK1 negatively regulated sporangiophore branching and sporangial production. PlBZP32 also negatively regulates the production of sporangiophores and sporangia, and belongs to a group of transcription factors that are usually regulated by various signaling pathways [39]. We speculated that PlRACK1 may act on signaling pathway proteins that activate the transcription factor PlBZP32, thus completing the negative regulation of sporangiophore and sporangial production. PlRACK1, PlMAPK1, and PlBZP32 all negatively regulated the branching of sporangiophores. PlRACK1 interacted with PlMAPK1 directly, suggesting that they may form a protein complex to regulate the signaling pathway of sporangiophore branching mediated by PlBZP32.

The growth of oomycetes requires resistance to stresses during normal development, and to overcome various environmental challenges [42,43]. The *PlRACK*1 mutant was more sensitive to cell wall stresses, but more tolerant of high osmotic pressure than WT. As an oomycete, *P. litchii* has a cell wall composition different from fungi, and contains cellulose rather than chitin. Whether to maintain normal morphology or to generate a cell wall in a short period of time during the early stages of pathogenesis to withstand high cell turgor and complete host surface attachment, the normal cell wall synthesis process and the integrity of the cell wall play important roles [42]. In *Saccharomyces cerevisiae*, *Ustilago maydis*, and *M*. *oryzae*, knockout or silencing of *RACK1* homologs also result in reduced tolerance to cell wall stresses [44].

Deletion of *PlRACK1* almost abolished the pathogenicity of *P. litchii*, which was a far greater impact than on growth, indicating its crucial role in the pathogenic process. To further explore how PlRACK1 mediates the pathogenicity of *P. litchii*, oxidative stresses, and laccase activity were measured. Reactive oxygen species (ROS) burst is an effective defense mechanism for plants to damage the growth of pathogens when faced with infection [45]. Knockout of *PlRACK1* reduced tolerance to H_2_O_2_. PlRACK1 showed the ability to regulate peroxidase in *P. litchii* to scavenge ROS. Laccase can act as a virulence factor to assist in infection and protect the pathogen from damage, and knockout of the *PlRACK1* gene also reduced laccase activity of *P*. *litchii*. These results suggested that PlRACK1 may modulate pathogenicity via ROS scavenging and regulating laccase activity.

RACK1 has a conserved function in regulating pathogenicity [24]. In *M. oryzae*, the deletion of RACK1 causes a conidial appressorium to be limited to infecting only one cell without timely invasion of adjacent cells by hyphae. RACK1 mutants of *M. oryzae* show a significantly decreased ability to infect rice, and RACK1 interacts with phosphodiesterase and regulates intracellular cAMP concentrations, thus affecting the pathogenicity of conidia [23]. Here, we further characterized the virulence function of PlRACK1 by transcriptome analyses. The differentially expressed genes in the transcriptomes of the WT and Δ*plrack1*-15 revealed that *PlMKP1* and *PlBglX* were down-regulated in Δ*plrack1*-15 and hence reduced the virulence of *P*. *litchii*.

PlMKP1 interacted with PlMAPK1 and PlMAPK2, which indicated that PlMKP1 may be involved in the regulation of the MAPK signaling pathway. MAPK phosphatases are important proteins responsible for regulating the dephosphorylation and inactivation of MAPK [46] and can maintain the stability of intracellular MAPK concentrations [47]. Therefore, these results provided experimental evidence for RACK1 and MKP associating with the MAPK regulatory network in an oomycete. The positive regulatory effects of PlRACK1 on PlMKP1 expression may function by deactivating PlMAPK proteins through the dephosphorylation effect of PlMKP1 on PlMAPK proteins to return PlMAPK proteins to their normal intracellular concentrations. Verification of the interaction of these proteins in *P. litchii* and determination of their enzymatic activities are still needed to demonstrate this regulatory network.

Knockout of *PlMKP1* significantly reduced the pathogenicity of *P*. *litchii*, which is consistent with the experimental results with the *PlRACK1* knockout mutant. Furthermore, knockout or silencing of PlMAPK2 and PlMAPK10 result in a significant decrease in pathogenicity [27,38]. We speculated that for *P. litchii*, both activation and deactivation of MAPK proteins play important roles in pathogenicity. In *M*. *oryzae*, MKP not only negatively regulates the activation of MAPK but also is essential for its pathogenesis [48]. Currently, most studies focus on the activation of MAPK proteins, and there is little research on the deactivation of MAPK proteins in pathogenic fungi and oomycetes; further studies are needed to reveal these mechanisms and effects.

In the closely related *P*. *sojae*, PsRACK1 regulates its sexual reproduction process without participating in the pathogenicity and asexual reproduction processes [49]. This is different from the functions of PlRACK1, which mainly involved pathogenesis and asexual production, suggesting that RACK1 homologs have different functions in different oomycetes.

This study reported the interactions and functions of RACK1, MAPKs, and MKP in oomycetes. PlRACK1 is also involved in pathogenicity of *P*. *litchii* via regulating *MAPKs* and *PlBglX* expression. These results provide new insights into the mechanism of action of RACK1 and MAPK signaling pathways in oomycetes, and lay the foundation for future research in this area.

## Supplementary Material

FigureS3.jpg

Table S2 The gene sequences used in this study.doc

FigureS8.jpg

FigureS6.jpg

Figure S1.jpg

FigureS4.jpg

Table S1 The primers used in this study.doc

Figure S2.jpg

FigureS7.jpg

FigureS5.jpg

## Data Availability

The data generated during the study is available at Harvard Dataverse at https://doi.org/10.7910/DVN/FTLPQK.
