# Supplementary figures and images for: A RACK1 family protein regulates pathogenicity of Peronophythora litchii by acting as a scaffold for MAPK signal modules
Source: Virulence. 2025 May 13;16(1):2503429. doi: 10.1080/21505594.2025.2503429 (PMC12077431; doi:10.1080/21505594.2025.2503429)

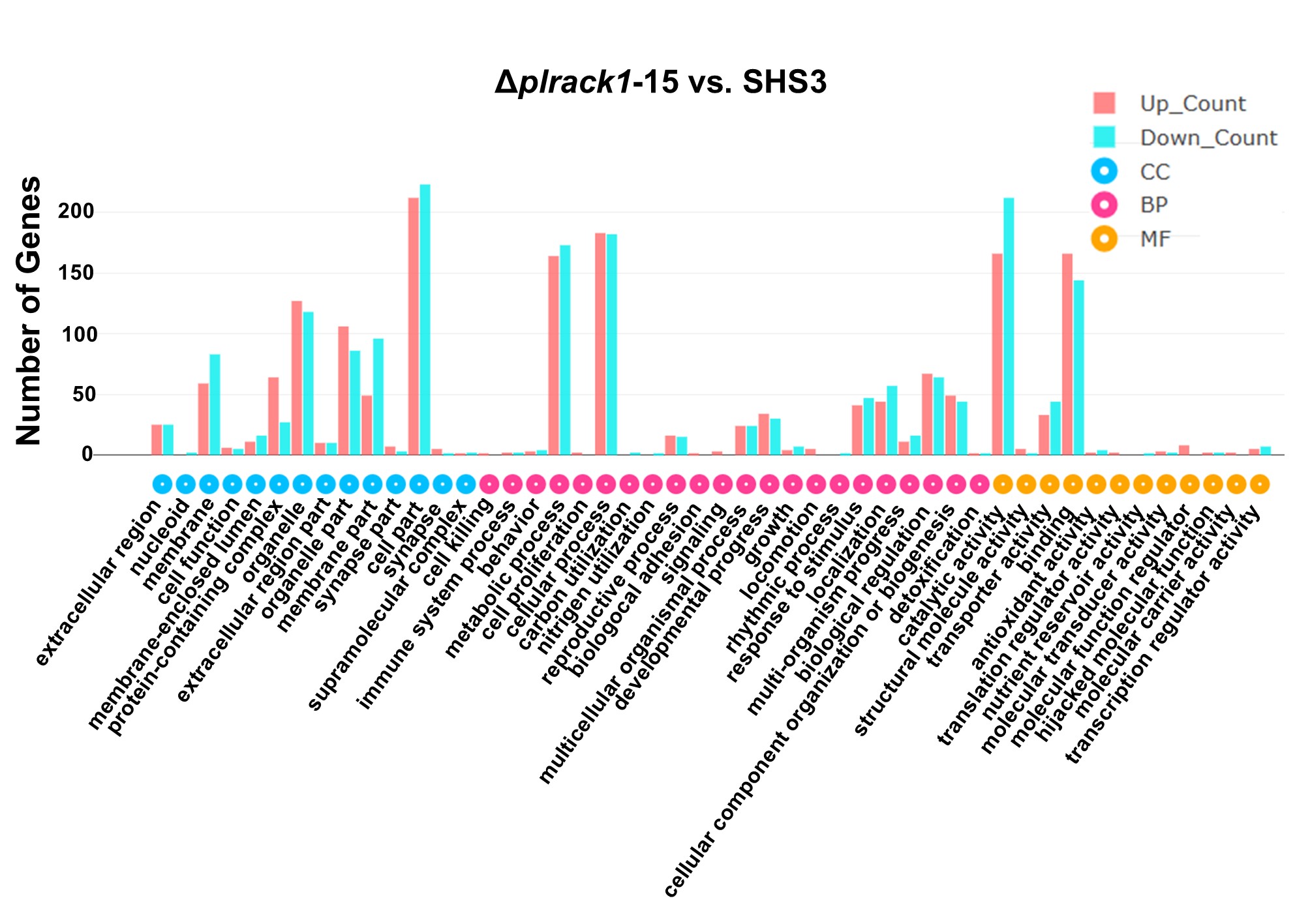

Supplement: FigureS3.jpg [file KVIR_A_2503429_SM5565.jpg]

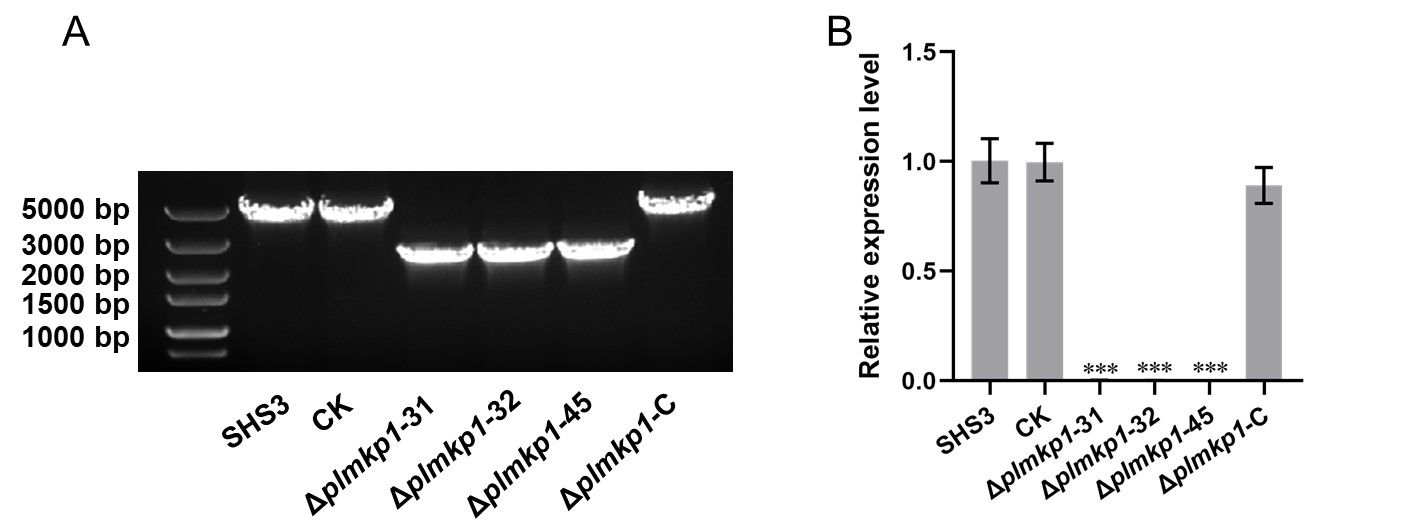

Supplement: FigureS8.jpg [file KVIR_A_2503429_SM5563.jpg]

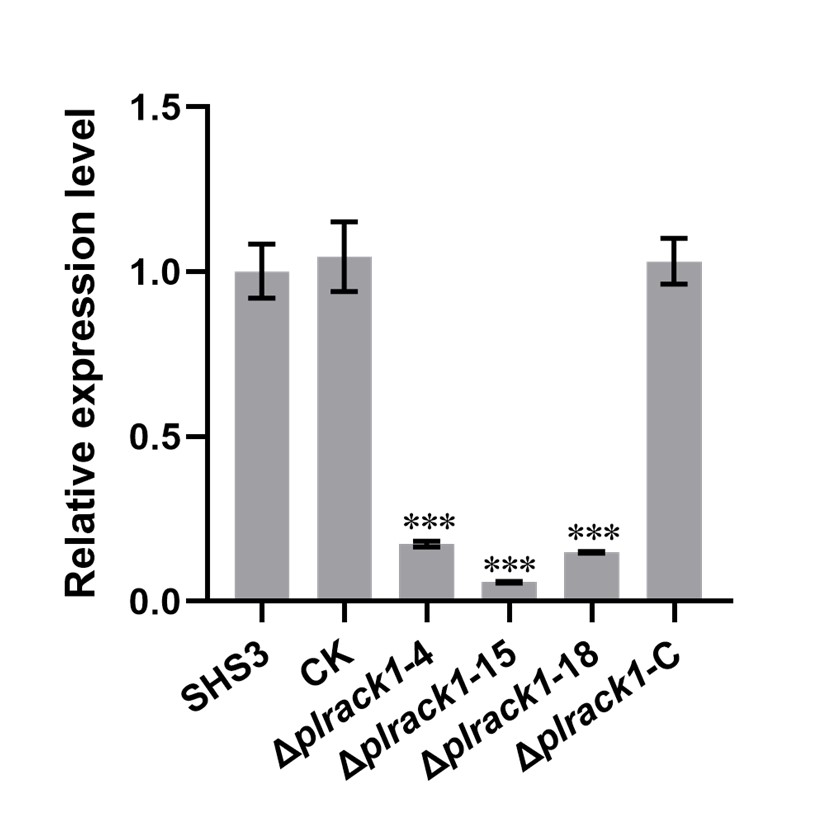

Supplement: FigureS6.jpg [file KVIR_A_2503429_SM5562.jpg]

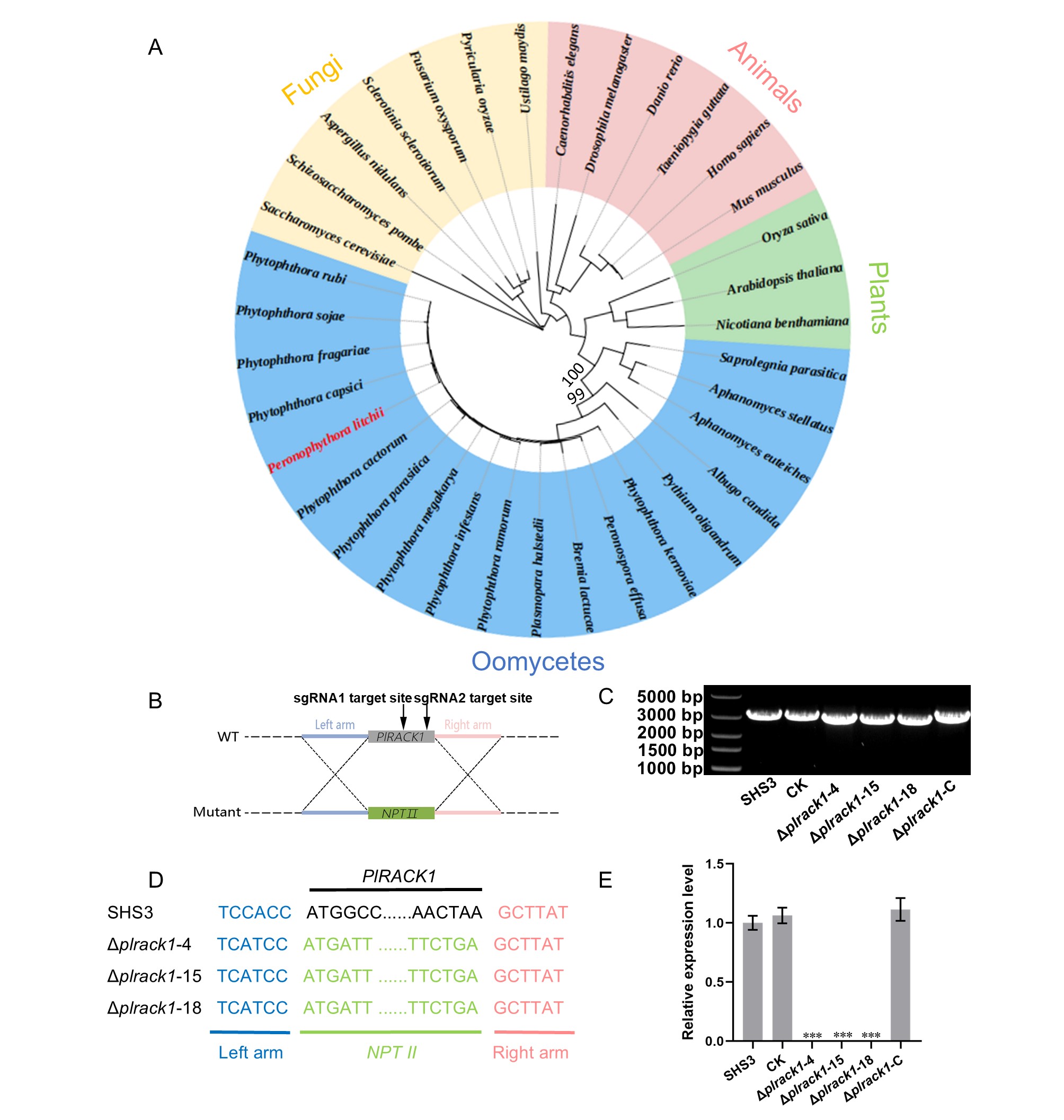

Supplement: Figure S1.jpg [file KVIR_A_2503429_SM5561.jpg]

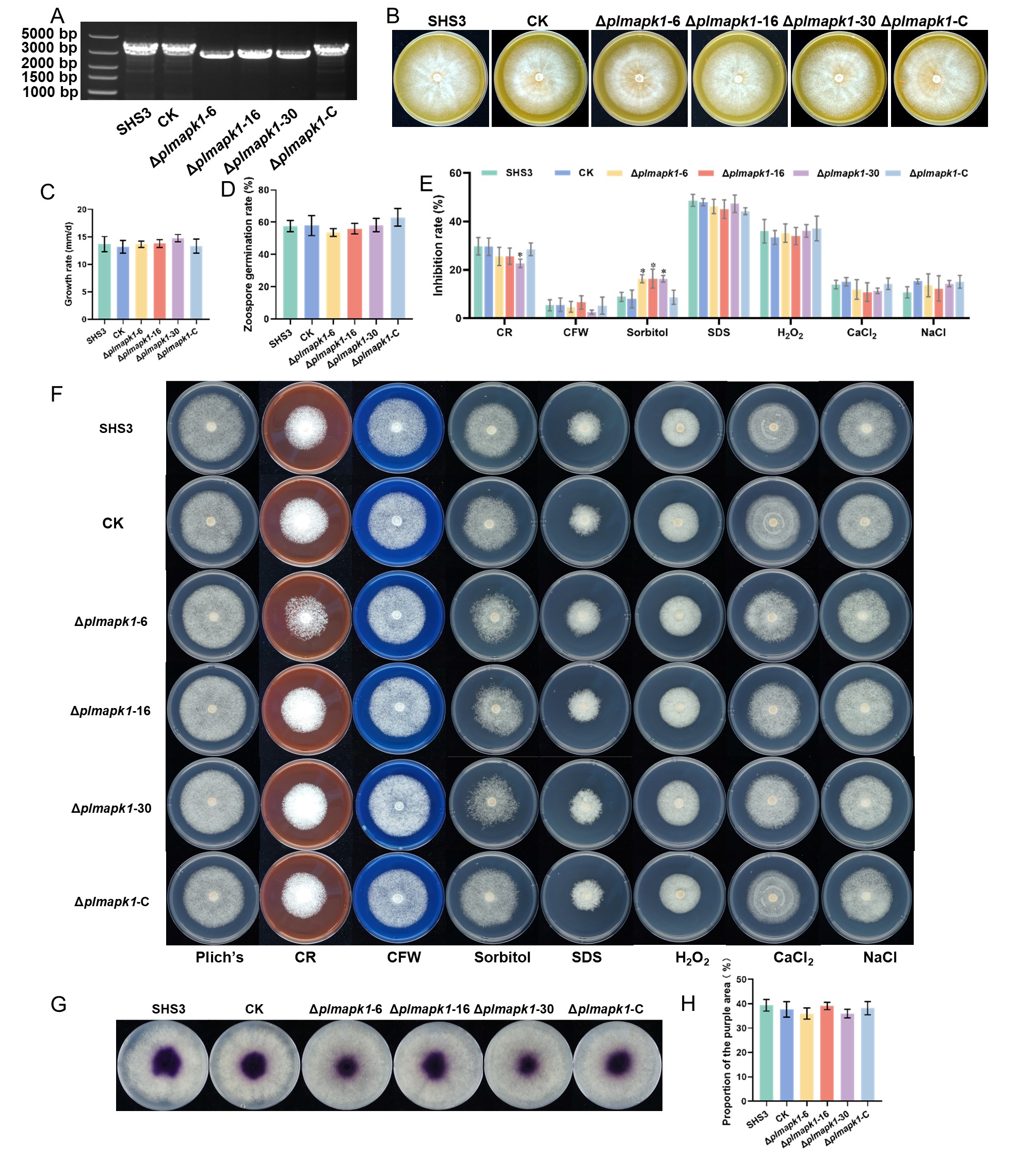

Supplement: FigureS4.jpg [file KVIR_A_2503429_SM5560.jpg]

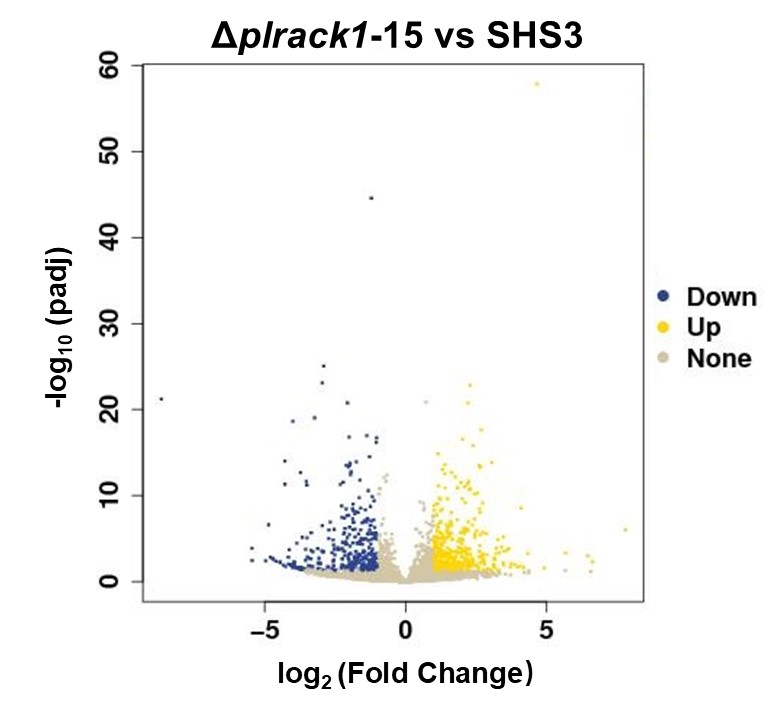

Supplement: Figure S2.jpg [file KVIR_A_2503429_SM5558.jpg]

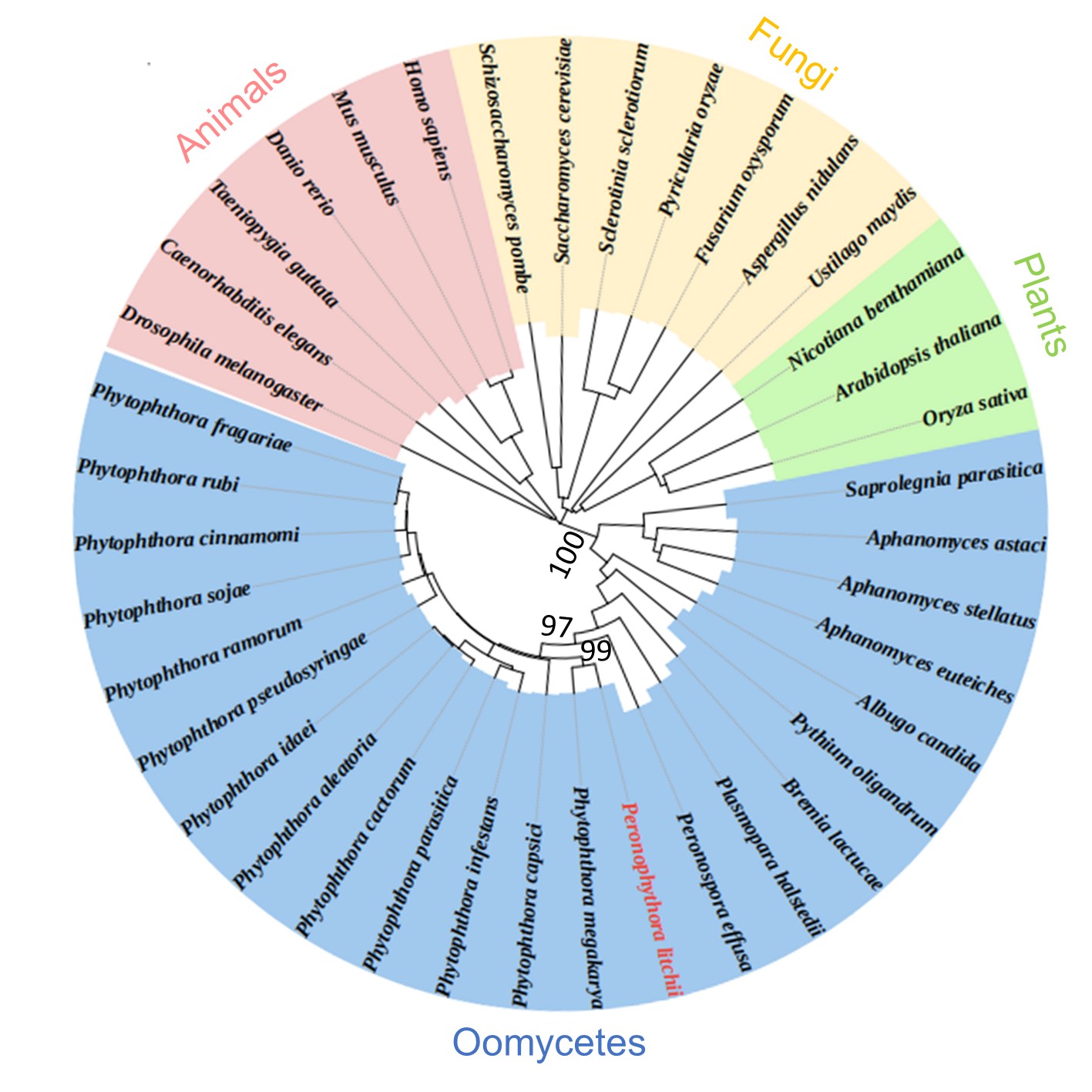

Supplement: FigureS7.jpg [file KVIR_A_2503429_SM5557.jpg]

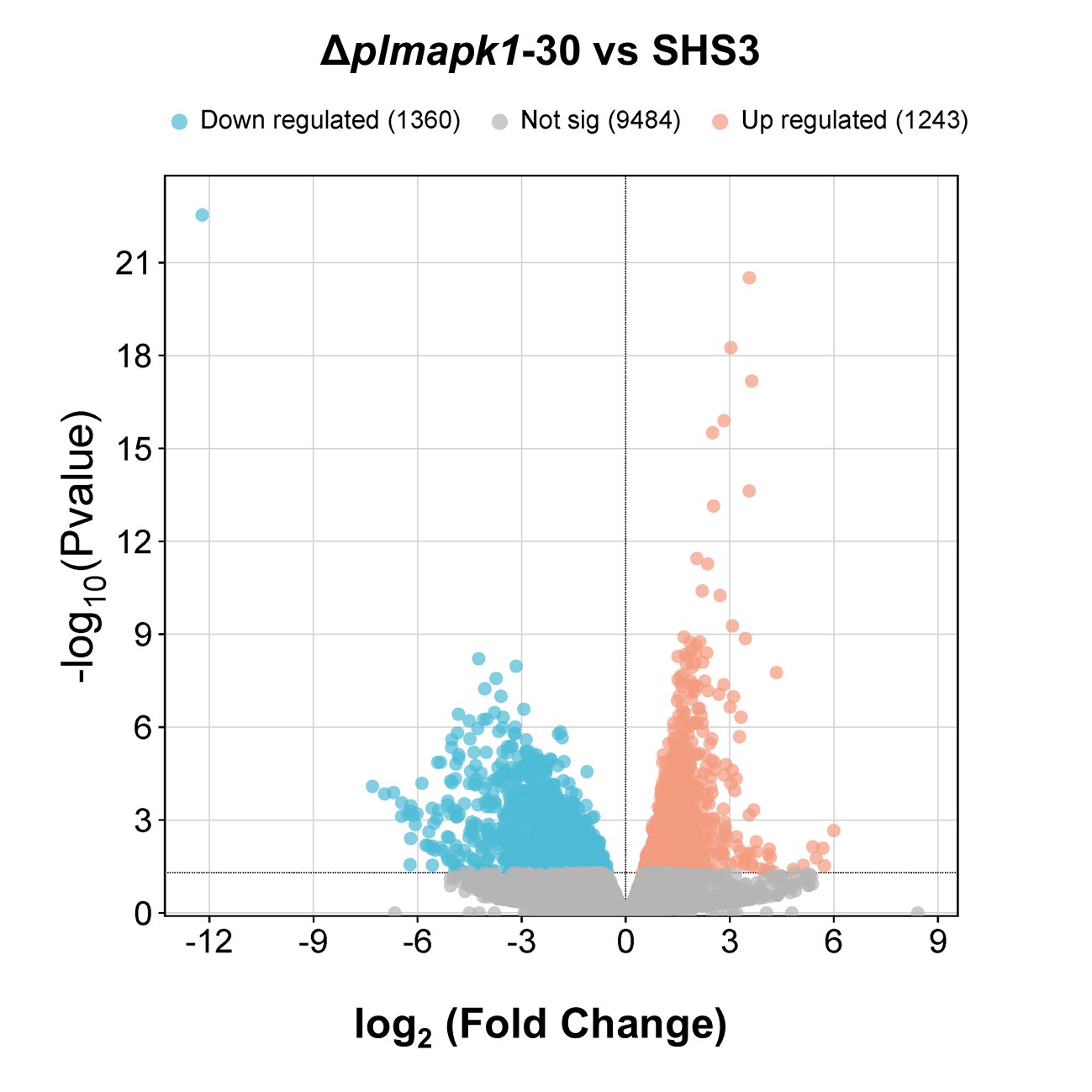

Supplement: FigureS5.jpg [file KVIR_A_2503429_SM5556.jpg]
