# Supplementary material for: A RACK1 family protein regulates pathogenicity of Peronophythora litchii by acting as a scaffold for MAPK signal modules
Source: Virulence. 2025 May 13;16(1):2503429. doi: 10.1080/21505594.2025.2503429 (PMC12077431; doi:10.1080/21505594.2025.2503429)
Supplement: Table S2 The gene sequences used in this study.doc [file KVIR_A_2503429_SM5564.doc]

Supplemental material Table S2. The gene sequences used in this study.

| Gene name | 5’ -3’ |
| --- | --- |
| PlRACK1  (*P. litchii* 007008) | ATGGCCGAGACCAGCATCTACCCGCGCGCCGAGCTTGACGGCCACAACGGCAAGGCCGTGACGGCCATCGCCACGACCCGCGAGAACCCGAACCTGCTTCTGACTGCCTCGCGCGACAAGTCGCTACTTGTGTGGCAGCTCTCGAACGACGGCGAGGAGTACGGCTTTGCCCGTCGTCGTCTGCAGGGCCACTCGCACTACGTCGAGGACGTGGTGATCTCGTCGGACGGTCAGTTCGCTCTGTCCGGCTCGTGGGACGGCACGCTACGTCTGTGGGACCTGAACACGGGCATCACGACGCGCCGCTTCGTGGGACACACCAAGGACGTGCTGTCGGTGGCTTTCAGCGCTGACAACCGTCAGATCGTCTCGGGCTCGCGTGACAAGACCGTGAAGCTCTGGAACACTCTGGGTGAGTGCAAGTACACCATTACGGAGGACGGACACACGGAGTGGGTCTCGTGCGTGCGCTTCAGCCCGTCGACGGCCAACCCCCTCATTGTCTCGTGTGGCTGGGACAAGGTCGTCAAGATCTGGAACCTGTCCAACTGCAAGCTCCGCACCAACCTGTTCGGCCACGAGGGCTACCTTAACACGGTCACAGTGTCTCCTGATGGATCGATCTGTGCTTCGGGTGGTAAGGACGGCACTGCCAACCTGTGGGACCTGAACGAGGGCAAGCGCCTCTACTCGCTTGTGGCTGGCGATGTCATCCACGCTCTCGTGTTCTCGCCCAACCGTTACTGGCTGTGCGCTGCCACCACCTCGGGCATCAAGATCTGGGACCTAGAGTCGAAGATCGTGGTGCACGACCTGCAGCCGGAGGTTGAGGAGCCCAAGGGCAAGTACGCTCAGCCGCCTCACTGCATCTCGCTGGCCTGGTCCGCTGACGGCTCGGTGCTCTTCTCGGGCTACACGGACGGTATCGTCCGCGTGTGGTCGGTGGGCAACTAA |
| PlMAPK1  (*P. litchii* 008047) | ATGTCGATCGAGCCATCTCGCTACGGTCCGGATTTCCACTGCGTCACTGTGTCCCGAGATGTCTTCGAAGTCCGTTCACACTACGTGAACTTGCGTCCAGTTGGCGGAGGTTCCTACGGCATTGTCTGCTCCGCTGAAGACACGCTACGAGGACGCAAAGTGGCCATCAAGAAGATCACCGACGTGTTCGACGATCTCACAGATGCCAAGCGGATCTTACGTGAAATGAAGCTGCTCCGACACCTCGGAGTGCATGAAAACATCATCAACATTCTGGACGTGATCTTGATTCCGCCCAATGTCATGGATTTCCACGACATCTACATTGTCACGGATCTTATGGAGAGCGACTTGGAGCGGATCATCAGCTCGTCACAACCCCTTTCGGATGCACATTTTCAGTACTTTTTGTACCAGATTTTGCGCGGTATGAAGTTTGTGCACTCGGGGAATGTGCTACATCGAGATCTCAAGCCGTCAAACTTGCTTGTGAACTCCAACTGTGATTTATCCATTTGTGACTTTGGTTTGGCGAGAGGTGTCGAGACGGCGCATAATGAAGATCTGACAGAGTATGTTGTCACACGATGGTATCGAGCACCGGAACTGCTCACGGATTGTCAAAACTACAACGATGCTGTGGACGTCTGGGCTGTTGGCTGCATTTTTGCAGAAATGTTGCGTCGTCGACCGTTCTTCACAGGACGAGACCCGTCAGATCAACTGCACATGATCATCCGAGTGCTAGGCTCACCTACAGAAGAAGAAATGGCCTTCGTACCACATGAAGCTGCGAAGCGCGCCATCTTGCAACATGGATTCTACCCAAAGCGCCCGTTGATCGAGTTCTTCCCAGATGCCAACCCACTGGCAGTGGATTTGCTCTCTCAAATGCTCAAATTCAATCCGGCTGAGCGTATTTCCGTCATGCAGGCCTTAGCACATCCGTACTTGGCGCAACTTCAGAATCCTGCCGACGAGCCCGTTTGTGCCGAGCCCTTCAACTTCGATTTCGAGCGCGAGTCGCTGGATTTGGGCGTCGAGATGCCCAAGGAGGAACTGCAGCGATTGGTCTTCCAGGAGTGCATGTCGATTCACCAGATAGAAGCGCATCATATGCAGTAA |
| PlMAPK2  *(P. litchii* 14182) | ATGGCCAGCTACACTCCGACCGGACCTGGTTCCTCCTCTCCATTGAAATCGATCGGTGGTCCCTCAGATACTTCGGCACCAGTTGGTGCAGTGAGTTCAACGCGTAAAGCGAGTACCACCACTGCAGTCGCCGCCCCTCCTGCTCGACCAGGCACGTACTCTTTTGTTGTAGCAGGAACAAATTTCCAGATCGACGACAAGTACAAATTTATCAAGGTCATTGGTCGTGGTGCTTATGGAGTTGTCATTTCGGCAGACAACGCCGAAACAAACGAAAAAGTAGCCGTAAAGAAAATATCGCGGGCTTTTGAAGATCTCGTGGATGCCAAGCGCATCTTGCGCGAGATTAAGCTCTTGCAGCATTTTGATCACGAAAACGTGATCACAATTGTCGACTTGTTGCCACCACCGTCCTTGGCACAGTTTGAAGACGTTTACATCATCGCAGATCTCATGGAGACCGACTTGCACCGAATAATCTACTCGAGGCAGCCGCTCACGGACGATCATGTGCAATACTTCCTGTACCAGATCTTGAGAGCACTCAAGTATATCCACTCGGCGAATGTCCTCCACCGAGATTTAAAACCGTCGAACTTGTTGTTGAACTCGAATTGCGACTTGAAAGTGTGCGATTTTGGCTTGTCGCGAGGTGTCGAGCCCGAGGAAGACAACATGGAGCTCACAGAATACGTCGTCACGCGCTGGTACCGCGCGCCTGAGATCATGCTTTCCTCGAGAGAGTACACCAAGGCAATTGACATTTGGTCCACAGGGTGCATTTTTGCAGAGCTACTCGGTCGCACACCACTTTTCCCTGGTGATGACTATATTCACCAACTACAGATCATTTGCGACAAGATCGGAACTCCATGTGAGGAAGACCTGCATTTTGTTGTCAGCGAGAGAGCTAAACGGTTCATGAAAAACCAACCTATGCGGCCTGGAGTGCCATTTGCCAAACTTTTCCTGAAAGCTTCTCCCGAGGCCATCGACTTGCTACAGAGCATGCTTGTGTTTGACCCAGCAAAACGGATCTCGGTGGAGGAAGCACTAGAGCACCCATACTTGGCATCTCTGCACAACCTGGAGGACGAGCCTGTAGCGGACAGCGCATTCAGTTTCGACTTCGAGAAAGAAGATCTAACGGAGTCTCGGTTGAAAGAGCTTATCTTCGAGGAGATCCTCAAAATTCATCCAGATGCACCGCGTAGCCCCTTAAAGATGCCTCATGCCGGGTTAAACAGCCCTCCGACAGAGCAAGTGCTTAGCCCAAGCACGACACGCTCGCACGCTCAGGACTAA |
| PlMKP1  *(P. litchii* 013853) | ATGGAACTCGCTCCTGTGGCCAGAACCAGCGAAGGCTCCAACGCCAAGCGGAAACTCGACACTGCACACACCATCTTCTCGTTCTCGGCGTCGTCGTCGCCCTCGGGGTCTCCTACTACCTCGTCCAACAGCTGCAAACCATTACGAGCCACCACGGACGCTCTACCTCCACTGGATTCGCACTCGAAGGTGCGACGTCGTGCTACTGCAGGGCCTCCGTCGTTAACGCGCTGTTACTCGGACGGGAAAATTTCAAAGGCGTTCGGAGAGTTGCTGACTGCAGAAGAAGTGGCAGACGGACAGAGACTACAACATGCCGCTCAAGTCTGTCCTACGTCACCGGCGCTGACGCCGCAGAAGAAGAATGTGTGTCCATCGCCCGGAATGCACCGCAATCCGTTCCTGGATGAGGAAACATGGCCATTGAGCGTTTCAAGAGGTCCGACGTCGACATCACCAGCATTTGTCTCCAAGAGCTACAACAACAACACGACTCCAACGCGGAACACGGGTGACAGCTGGGCCATGTTTACTCCACCGACGCCGAACAAGCGAGTTTGCAGCCAGCAGGGTTTTACACAGAGCAAGGCAGGTGCGACTGCTTCACCGGGTAACTGGGCGCCTTCTCTTACACAAGCAAGAAAGCGCTCTCTGGGGGTGTCTGATGCCGCTCACTTCGCTAGTGCGTTGCCGACAGACAAGCGAACGTCGTTGTCCATCGACATGAATTTAGAGAGCCGAAGTAATGCTGCCGGGTCACAGTCGTCGTGTTCGTCGTCATCGATTCCTTCAGTCACCGCTTTACCCCGAGGTAGTGTGAGCGCGCCGAAGAGAATGTATCCGCCTCAGCAACGCCGGAGCCGGAACCCAGGCAACTTGTCGTTGGACTTATCGCAGGTAGATCAGACGAACTTCGATCCGTGCACAACTACAACCGGTGCAACGAAGCGGCGGAATAACCAAGCTGCTGTGTGCTCGAAGATTACGGACTTCCTGTACATTGGTGGTGCTGTAGCTGCTAAGAACAAGTCGATGCTGATCCAGAATGGCATTACGCATGTCATTAACTGCGCTGCCAGTGTGGCTCCAGCCTCTTTCCCGGATGAGTTCTGCTACTTCAACATCAGACTCCGCGATCATTCATCGCAGGACATTGCACGACACTTTTACAGCATGTTCGACTTCATCGAGCGTGCTCGTGAATGCGGCGGACGAATATTTTTGCACTGTGTCAAAGGCATTTCTCGCTCACCTACCATGGCAATCGCGTATTTGATGTGGTACAAGCATATGGGCATGTACAAAGCTTTGGACTTTGTGCGTCAGGCACGTCCAATTGTAGACCCGAATGCTGGTTTCATCTTTCAGTTGACTGAGTGGGAACAAGTTCATCCGGAAGGCAGACTCAAATTCCAGCGTACTATCATCTTTCGAATGGATGTGGCGTATGCCAACCCCGACAAGAGTGGCAACTGCAACAACTTTGTAGCTGAAAAGAACCCCCTGTTTGTAGGCCCCCTTCCGGGTATCAGCGAGAACTACTTCCGTGATCCAACCAAGGATATCGGTGAGTTATGCTTGATCGTGGCATGCGCCGACTATATGTTCGTATGGTGTGGCACCGACGTCAATGGTGACCAAGTGGAGGTTGGCGAGAGTGGAGCGCAGATCCTGCAGCGATATGAAGGGTTCCCAGCCAAGTGCGATACTGTGCGGCAAGGACAGGAGCCTGTTGCTTTCTGGGACCTCGTTGGTGACGAAATTTGA |
| PlBglX  (*P. litchii* 000214) | ATGGTCTCCGTCACATGGGGCATCGCATTAGCACTATGTACGCAACTTGTCAGTGCTGGCGACCCGCTCGCCACCGTTTCCTGGCGTACAGGAAACGACAAGGTCGACGCCATCGTCGAGGCCCTGACGGCTGAAGAAAAGGTCAGCCTCGTTCACGGCGCTGTTGATTCAGATGGCAACCAACAGCAAGCCGGCTACAGCGTCCCGATCACGTCGCAGGGCATCCCCGCTATCCGTCTCACGGACGGTGAAGCCGGCATCAACATCGTGCAGAACGCCACGGGTCCACCCACCCAGCTCAACGTCGCTGCCACTTGGTCATTGAACGCCGCATACCAGCACGGTGTGATCACAGGCAAGGAGGCGAAGCTGTTCAACATGGCGGTAGCTCTATCGCCTCGCGTTAACATCCTGCGTGATCCTGTAGAAGGTAACTTTTGGCAGTCGTACAGCGAGGACCCGTTCCTGAATGCTCGTCTCGGTATTCAAGGCGTGAAAGGCCTCCAGGACCAGGGCTCCATGGCCAACGCCAAGCAGATCGGTCCGTCATCGACCGGTGCCAGTGCTGGTGATGACAACAGCGTCGTTGATTTGCAAACACTACAGGAGCTGTATTGGGTTGCCCCTGGTACCCTTCTGCAGGAAGGCAATGCTGCCACGCTCATGTGCTCATATGCGCAGGTTAATGGCATCCCTGCATGCCAGTACGAGCCGCTATTCAACACGATGCGCGACGACTACAACAGCTCCGCCATTGTCATGAGTGACTGGGGTGCGACGCACTCGACCGCCGAAAGTTTGATCGCCGGTATGGATCTGGAGATGCCGACGGGTTCGTACTACGGCGCGCTCTACGAATACATCTACGTCGCACAGAATCTGAGCGAGTCGTACTTGAATCGCGCCGTAGGTCACATCCTGGCCAAATACGACGAATTCGGACTGCTAGGAACAACCACTGAGGGTTCATCGCCGCTGTCGCAGGATGTGATCGAGGACCACGCACAGATCTCGTACGACATCGCCGTCAAGTCGGGCATCCTGCTCAAGAACACGAACGACACGCTGCCCATCAAGTCGGGATCTTCGATCGCTGTGATCGGCCCCAACGGCGTGCAATACACGCACGGTACGAACTTCGCAGAGCGTGCGTACGGCATCCCGGAACGTCAGATTTCAGTGCTTGAGGCTCTGAAATCTCGTCTCGGTGAGGATGTCGCGAACACCGTCGGTGTGGACCAGGAGGGCACAATTATCCCATCTACCCACCTGCGTAACCTGCAGGGCAACCCTGGATTGAGTCGTAATGACACCTTGGGCGGCACCTCGAACGACGAAATCGTGTACTTCACAGGCACGTCTGCGCTGGCTAAGAACGCGTCGTACACGTGGCAAGGACAAGTGCATGCGGAGACGGAAGGCTACTACACGTTTTCATTCGCCCGCGCCATCCCGAACTGGGAAAACCACACGAACCCGGACTACGGCTCCATTTTCGCTATCGGAACATTCAGCATCAACGGCACGGAGGTTGGTGAAGGCTACCGTCTCTACGGCGACGGTGGTGTCAAGCCCTGGAGTAACTCGATCGCCACTCGCGACAACTGGGACAATATCAAGTCGTACGTGTACCTGGGGGAAGGCTGGCACGACCTGGAGGCGTACATCGTGGGTCTCATCGACGAGCCGACGTCCGTGCGCTTGACTTGGGTGACGCCCACTCAGCGCGCAGCTAACATCCAAACTGCAGTTGATGTCGCCAAGGAGGTCGACACACCCATCGTGTTCGCGTTCGCCAACAGCCCTGCCCAAACGGGTTTGACGCTGGACGACGGTCAGGACGAGCTGGTAACGAAGGTGGCTGCTGCGAACCCGAACACTGTGGTGGTGCTGAACAACGCCGAACCTGTGTTGATGCCTTGGATCGACGACGTGGCTGCCGTGCTGGAGATGTTGTACCCGAACCAGGAGGGAGGTTGGGCCACTGCCGACTTGTTGCTGGGCAACAACGTGCCGCAAGGTCGGCTGCCAGTGACGTACCCGGCGAACTTAGACTCGACGATGACCCGTAACCCCGACTACCCCGAGCGCGTGGCTACTGAAAGCGGCAACGCGACATTCAGTGAGGGTTTGAACAACGGATACCGCTGGTACACACACACGAATACGAGCGTGTTGTTCCCATTCGGCTACGGTTTGAGTTACACGTCGTTTGAGTACAGCAACCCAAACTTCGCGGCATCAAGTTCCGCCCAGCAGGGCAGCAACGGCCTGAATTACGAGGTGTCATGCAGCCCAGACCACGCTGACGTCGTCTTCACTGTGTCCTTCACGGTCACGAACACTGGGAGTGTGAAGGGCGTGGAGGTGCCGCAGGTGTATATCGGCCCACCGGTAGATGGCGCGACGACGTACCCGGATGTACAGTTCGCTGCAACGGCACTGGTAGGCTTCGCGAACGTAGAGCTGGAGGCTGGTGCTAGTACGACCGTGAGCATCGGCGTCCTCGAGAAGCAGTTGAGCTTCTATAATGTAAACACCACGAGCTGGGAGGTAGCGAAGGGTGAACGTCCCGTGTACATCAGCAAGAATGCGGAGGACGCGGTGTTCAGCGGCCGCGTGCAGATCTAA |
