# Supplementary material for: A RACK1 family protein regulates pathogenicity of Peronophythora litchii by acting as a scaffold for MAPK signal modules
Source: Virulence. 2025 May 13;16(1):2503429. doi: 10.1080/21505594.2025.2503429 (PMC12077431; doi:10.1080/21505594.2025.2503429)
Supplement: Table S1 The primers used in this study.doc [file KVIR_A_2503429_SM5559.doc]

Supplemental material Table S1. The primers used in this study

| Primer name | 5’ -3’ | Usage |
| --- | --- | --- |
| pET32aPlRACK1F | TGGCTGATATCGGATCCATGGCCGAGACCAGCATC | Cloning PlRACK1 into pET32a vector |
| pET32a PlRACK1R | ACGGAGCTCGAATTGTTGCCCACCGACCACAC | Cloning PlRACK1 into pET32a |
| pET32aF | CACGCGGTTCTGGTATGAAAGAAA | sequencing primers for the pET32a vector |
| pET32aR | CTTTGTTAGCAGCCGGATCTCAGT | sequencing primers for the pET32a vector |
| pGEX PlMAPK1F | CTGGGATCCCCGGAAATGTCGATCGAGCCA | Cloning PlRACK1 into pGEX-6P-1 vector |
| pGEX PlMAPK1R | TCGACCCGGGAATTACTGCATATGATGCGC | Cloning PlRACK1 into pGEX-6P-1 vector |
| pGEX PlMAPK2F | CCTGGGATCCCCGGAATTCATGGCCAGCTACACT | Cloning PlMAPK2 into pGEX-6P-1 vector |
| pGEX PlMAPK2R | CTCGAGTCGACCCGGTTAGTCCTGAGCGTGCGA | Cloning PlMAPK2 into pGEX-6P-1 vector |
| pGEXF | AGGGCTGGCAAGCCACGTTTGGTG | sequencing primers for the pGEX-6P-1 vector |
| pGEXR | TCCGGGAGCTGCATGTGTCAGAGG | sequencing primers for the pGEX-6P-1 vector |
| PlRACK1  outF | ATCCAGCTACTGCACCTAATC | Screening the knockout and complementation mutants of PlRACK1 |
| PlRACK1  outR | CATTCACTCTAGCAGCGGAG | Screening the knockout and complementation mutants of PlRACK1 |
| PlRACK1 qPCRF | GACCGTGAAGCTCTGGAACA | Primer for qPCR of PlRACK1 |
| PlRACK1 qPCRR | AGCTTGCAGTTGGACAGGTT | Primer for qPCR of PlRACK1 |
| PlRACK1 LF | ACTAGTGGATCCCCCCATCGTCTTACTTGGTTTGAAC | Cloning of RACK1 left arm |
| PlRACK1 LR | ATCTTGTTCAATCATGGATGAAGAGATTTTCCAAAGTG | Cloning of PlRACK1 left arm |
| PlRACK1 RF | GACGAGTTCTTCTGAGCTTATCATCGAACTTCGTTGG | Cloning of PlRACK1 right arm |
| PlRACK1 RR | GAATTCCTGCAGCCCCCAAGTGTTGAGACCGAGAC | Cloning of RACK1 right arm |
| PlRACK1 sgRNA1F | CTAGCCACACGCTGATGAGTCCGTGAGGACGAAA  CGAGTAAGCTCGTCCGTGTGTCCGTCCTCCGTAA | Synthesizing the sgRNA for knocking out PlRACK1 |
| PlRACK1 sgRNA1R | AAACTTACGGAGGACGGACACACGGACGAGCTTA  CTCGTTTCGTCCTCACGGACTCATCAGCGTGTGG | Synthesizing the sgRNA for knocking out PlRACK1 |
| PlRACK1 sgRNA2F | CTAGCCCGTCACTGATGAGTCCGTGAGGACGAAA  CGAGTAAGCTCGTCTGACGGCTCGGTGCTCTTCT | Synthesizing the sgRNA for knocking out PlRACK1 |
| PlRACK1 sgRNA2R | AAACAGAAGAGCACCGAGCCGTCAGACGAGCTTA  CTCGTTTCGTCCTCACGGACTCATCAGTGACGGG | Synthesizing the sgRNA for knocking out PlRACK1 |
| pET32a PlMKP1F | GTCGACGGAGCTCGAATTAATTTCGTCACCAACGAGG | Cloning PlMKP1 into pET32a vector |
| pET32a PlMKP1R | ATGGCTGATATCGGATCCATGGAACTCGCTCCTGTGGC | Cloning PlMKP1 into pET32a vector |
| PlMKP1LF | GAACTAGTGGATCCCCCGCCTCCAGCTCAGCAAG | Cloning of PlMKP1 left arm |
| PlMKP1LR | CTGATGTACTATACATTATGACGTCTTGCGATTGATG | Cloning of PlMKP1 left arm |
| PlMKP1RF | ATCAATCGCAAGACGTCATAATGTATAGTACATCAGTT | Cloning of PlMKP1 right arm |
| PlMKP1RR | ATCGAATTCCTGCAGCCCAGTTTTCTTCGGGCTGTTC | Cloning of PlMKP1 right arm |
| PlMKP1 qPCRF | ACGCTCTACCTCCACTGGAT | Primer for qPCR of PlMKP1 |
| PlMKP1 qPCR R | TAGTCTCTGTCCGTCTGCCA | Primer for qPCR of PlMKP1 |
| PlMKP1 sgRNA1 F | CTAGCACGTCACTGATGAGTCCGTGAGGACGAAA  CGAGTAAGCTCGTCTGACGTAGGACAGACTTGAG | Synthesizing the sgRNA for knocking out PlMKP1 |
| PlMKP1 sgRNA1 R | AAACCTCAAGTCTGTCCTACGTCAGACGAGCTTA  CTCGTTTCGTCCTCACGGACTCATCAGTGACGTG | Synthesizing the sgRNA for knocking out PlMKP1 |
| PlMKP1 sgRNA2 F | CTAGCACCCCGCTGATGAGTCCGTGAGGACGAAA  CGAGTAAGCTCGTCCGGGGTAAAGCGGTGACTGA | Synthesizing the sgRNA of PlMKP1 |
| PlMKP1 sgRNA2 R | AAACTCAGTCACCGCTTTACCCCGGACGAGCTTA  CTC GTTTCGTCCTCACGGACTCATCAGCGGGGTG | Synthesizing the sgRNA for knocking out PlMKP1 |
| PlMKP1 outF | ATCACGTAATGGCAAGCAAAAGGAGCAA | Screening the knockout and complementation mutants of PlMKP1 |
| PlMKP1 outR | CGACAATGCTTTCTTCATCCTCCGTAGC | Screening the knockout and complementation mutants of PlMKP1 |
| BD PlMKP1F | GGCCATGGAGGCCGAATTCATGGAACTCGCTCCTGTG | Cloning PlMKP1 into pGBKT7 |
| BD PlMKP1R | GCAGGTCGACGGATCCTTATCAAATTTCGTCACCAAC | Cloning PlMKP1 into pGBKT7 |
| AD PlMAPK1F | CATGGAGGCCAGTGAATTCATGTCGATCGAGCCATCTC | Cloning PlMAPK1 into pGADT7 |
| AD PlMAPK1R | CGAGCTCGATGGATCCTTACTGCATATGATGCGCTTCT | Cloning PlMAPK1 into pGADT7 |
| AD PlMAPK2F | CATGGAGGCCAGTGAATTCATGGCCAGCTACACTCCG | Cloning PlMAPK2 into pGADT7 |
| AD PlMAPK2R | CGAGCTCGATGGATCCTTAGTCCTGAGCGTGCGAGCG | Cloning PlMAPK2 into pGADT7 |
| PlActin qPCRF | TCACGCTATTGTTCGTCTGG | Internal reference primers for qPCR |
| PlActin qPCRR | TCATCTCCTGGTCGAAGTCC | Internal reference primers for qPCR |
| PlMAPK1 LF | TCTAGAACTAGTGGATCCCCCGACGTCGGAACCCCCACCATC | Cloning of PlMAPK1 left arm |
| PlMAPK1 LR | AATCCATCTTGTTCAATCATGATAGTGCACTTGATATGGG | Cloning of PlMAPK1 left arm |
| PlMAPK1 RF | TTCTTGACGAGTTCTTCTGAGAGCGGTTGATCGATTCCAG | Cloning of PlMAPK1 right arm |
| PlMAPK1 RR | ATATCGAATTCCTGCAGCCCATGGGGCTGTGCATCCGTCA | Cloning of PlMAPK1 right arm |
| PlMAPK1 outF | GAACGGACAGAGACCTCAAAAGCTC | Screening the knockout and complementation transformants of PlMAPK1 |
| PlMAPK1 outR | CGCATTTGCCGCGCTGCTGGATGC | Screening the knockout and complementation transformants of PlMAPK1 |
| PlMAPK1 sgRNA1F | CTAGCGCGCTCCTGATGAGTCCGTGAGGACGAAACGAGTAAGCTCGTCGAGCGCGAGTCGCTGGATTT | Synthesizing the sgRNA for knocking out PlMAPK1 |
| PlMAPK1 sgRNA1R | AAACAAATCCAGCGACTCGCGCTCGACGAGCTTACTCGTTTCGTCCTCACGGACTCATCAGGAGCGCG | Synthesizing the sgRNA for knocking out PlMAPK1 |
| PlMAPK1 sgRNA2F | CTAGCAACTACCTGATGAGTCCGTGAGGACGAAACGAGTAAGCTCGTCGTAGTTAGTAAGTTTGATAT | Synthesizing the sgRNA for knocking out PlMAPK1 |
| PlMAPK1 sgRNA2R | AAACATATCAAACTTACTAACTACGACGAGCTTACTCGTTTCGTCCTCACGGACTCATCAGGTAGTTG | Synthesizing the sgRNA for knocking out PlMAPK1 |
| PlMAPK1 qPCRF | TGCACATGATCATCCGAGTG | Primer for qPCR of PlMAPK1 |
| PlMAPK1 qPCRR | AGCCGGATTGAATTTGAGCA | Primer for qPCR of PlMAPK1 |
| NPTII-F | ATGATTGAACAAGATGGATTGCACG | Cloning of NPTII-F |
| NPTII-R | TCAGAAGAACTCGTCAAGAAGGC | Cloning of NPTII-F |
| PlBZP32 qPCRF | TTCACGTCGCCTAATGAGTC | Primer for qPCR of PlBZP32 |
| PlBZP32 qPCRR | GGTTTAGCCCTGGAACGTAG | Primer for qPCR of PlBZP32 |
| PlBglXLF | CTAGAACTAGTGGATCCCCCGTATACCTATACGAGAGTGA | Cloning of PlBglX left arm |
| PlBglXLR | CTTCACATTTGCTAGCTGTCGATTCCCGAACCGAACACTA | Cloning of PlBglX left arm |
| PlBglX RF | TAGTGTTCGGTTCGGGAATCGACAGCTAGCAAATGTGAAG | Cloning of PlBglX right arm |
| PlBglX RR | ATATCGAATTCCTGCAGCCCATCAGACCATGTCGTGTTCA | Cloning of PlBglX right arm |
| PlBglX outF | AGTTGTTGTAGGCAGTTAGGAC | Screening the knockout transformants of PlBglX |
| PlBglX outR | ATCACGATTGCCACCTTGATAT | Screening the knockout transformants of PlBglX |
| PlBglX sgRNA1F | CTAGCGACGGTCTGATGAGTCCGTGAGGACGAAA  CGAGTAAGCTCGTCACCGTCCGTGAGACGGATAG | Synthesizing the sgRNA for knocking out PlBglX |
| PlBglX sgRNA1R | AAACCTATCCGTCTCACGGACGGTGACGAGCTTA  CTCGTTTCGTCCTCACGGACTCATCAGACCGTCG | Synthesizing the sgRNA for knocking out PlBglX |
| PlBglX sgRNA2F | CTAGCGCCGGTCTGATGAGTCCGTGAGGACGAAA  CGAGTAAGCTCGTCACCGGCGATCAAACTTTCGG | Synthesizing the sgRNA for knocking out PlBglX |
| PlBglX sgRNA2R | AAACCCGAAAGTTTGATCGCCGGTGACGAGCTTA  CTCGTTTCGTCCTCACGGACTCATCAGACCGGCG | Synthesizing the sgRNA for knocking out PlBglX |
| PlMAPK1complementationsgRNA1F | CTAGCGCGAGCCTGATGAGTCCGTGAGGACGAAACGAGTAAGCTCGTCGCTCGCTTCGCTGCTACTTA | Synthesizing the sgRNA for complementation of PlMAPK1 |
| PlMAPK1complementationsgRNA1R | AAACTAAGTAGCAGCGAAGCGAGCGACGAGCTTACTCGTTTCGTCCTCACGGACTCATCAGGCTCGCG | Synthesizing the sgRNA for complementation of PlMAPK1 |
| PlMAPK1complementationsgRNA2F | CTAGCAAGGCTCTGATGAGTCCGTGAGGACGAAACGAGTAAGCTCGTCAGCCTTCTTTCGGCTGACGC | Synthesizing the sgRNA for complementation of PlMAPK1 |
| PlMAPK1complementationsgRNA2R | AAACGCGTCAGCCGAAAGAAGGCTGACGAGCTTACTCGTTTCGTCCTCACGGACTCATCAGAGCCTTG | Synthesizing the sgRNA for complementation of PlMAPK1 |
| PlRACK1complementationsgRNA1F | CTAGCCAGGGACTGATGAGTCCGTGAGGACGAAACGAGTAAGCTCGTCTCCCTGTACCAGGTAAATAG | Synthesizing the sgRNA for complementation of PlRACK1 |
| PlRACK1complementationsgRNA1R | AAACCTATTTACCTGGTACAGGGAGACGAGCTTACTCGTTTCGTCCTCACGGACTCATCAGTCCCTGG | Synthesizing the sgRNA for complementation of PlRACK1 |
| PlRACK1complementationsgRNA2F | CTAGCCAGTCACTGATGAGTCCGTGAGGACGAAACGAGTAAGCTCGTCTGACTGGTGGGTATCAAAAG | Synthesizing the sgRNA for complementation of PlRACK1 |
| PlRACK1complementationsgRNA2R | AAACCTTTTGATACCCACCAGTCAGACGAGCTTACTCGTTTCGTCCTCACGGACTCATCAGTGACTGG | Synthesizing the sgRNA for complementation of PlRACK1 |
| PlMKP1complementationsgRNA1F | CTAGCACCTGACTGATGAGTCCGTGAGGACGAAACGAGTAAGCTCGTCTCAGGTAAGAGCTGCACATA | Synthesizing the sgRNA for complementation of PlMKP1 |
| PlMKP1complementationsgRNA1R | AAACTATGTGCAGCTCTTACCTGAGACGAGCTTACTCGTTTCGTCCTCACGGACTCATCAGTCAGGTG | Synthesizing the sgRNA for complementation of PlMKP1 |
| PlMKP1complementationsgRNA2F | CTAGCGGTTGGCTGATGAGTCCGTGAGGACGAAACGAGTAAGCTCGTCCCAACCATCTCCACTGCATA | Synthesizing the sgRNA for complementation of PlMKP1 |
| PlMKP1complementationsgRNA2R | AAACTATGCAGTGGAGATGGTTGGGACGAGCTTACTCGTTTCGTCCTCACGGACTCATCAGCCAACCG | Synthesizing the sgRNA for complementation of PlMKP1 |
| PlBglXcomplementationsgRNA1F | CTAGCGTGATCCTGATGAGTCCGTGAGGACGAAACGAGTAAGCTCGTCGATCACCTCAAGATTTGCGT | Synthesizing the sgRNA for complementation of PlBglX |
| PlBglXcomplementationsgRNA1R | AAACACGCAAATCTTGAGGTGATCGACGAGCTTACTCGTTTCGTCCTCACGGACTCATCAGGATCACG | Synthesizing the sgRNA for complementation of PlBglX |
| PlBglXcomplementationsgRNA2F | CTAGCTCAACCCTGATGAGTCCGTGAGGACGAAACGAGTAAGCTCGTCGGTTGACGTTGAAGTGGTAA | Synthesizing the sgRNA for complementation of PlBglX |
| PlBglXcomplementationsgRNA2R | AAACTTACCACTTCAACGTCAACCGACGAGCTTACTCGTTTCGTCCTCACGGACTCATCAGGGTTGAG | Synthesizing the sgRNA for complementation of PlBglX |
